# MAZ mediates the cross-talk between CT-1 and NOTCH1 signaling during gliogenesis

**DOI:** 10.1038/srep21534

**Published:** 2016-02-12

**Authors:** Bin Liu, Anyun Ma, Feng Zhang, Yumeng Wang, Zengmin Li, Qingyu Li, Zhiheng Xu, Yufang Zheng

**Affiliations:** 1 State Key Laboratory of Genetic Engineering and Ministry of Education (MOE) Key Laboratory of Contemporary Anthropology, School of Life Sciences, Fudan University; 2 The Institute of Developmental Biology and Molecular Medicine, Fudan University, Shanghai, China, 200433; 3 State Key Laboratory of Molecular Developmental Biology, Institute of Genetics and Developmental Biology, Chinese Academy of Sciences, Beijing, China 100101; 4 Innovation Center for International Cooperation of Genetics and Development, Fudan University, Shanghai, China, 200433

## Abstract

Neurons and glia cells are differentiated from neural stem/progenitor cells (NSCs/NPCs) during brain development. Concomitant activation of JAK/STAT and NOTCH1 signaling is required for gliogenesis, a process to generate glia cells to ensure proper brain functions. NOTCH1 signaling is down-regulated during neurogenesis and up-regulated during gliogenesis. However, the underlying mechanism remains elusive. We report here that cardiotrophin-1 (CT-1) activates NOTCH1 signaling through the up-regulation of ADAM10, a rate-limiting factor of NOTCH1 signaling activation. We found that a transcriptional factor, Myc-associated zinc finger protein (MAZ), plays an important role in ADAM10 transcription in response to CT-1 in NPCs. MAZ knockdown inhibits CT-1 stimulated gliogenesis and it can be rescued by over-expressing human NICD. Our results provide a link between NOTCH1 activation and neuronal secreted CT-1, suggesting that CT-1 plays an important role in ensuring the coordinated activation of NOTCH1 signaling during gliogenesis.

Glia cells which account for more than 90% of cells in the brain, are essential for neuronal functions^1^. They not only provide nutrients but also regulate the activity of neurons and mediate synapse transmission efficiency^2^,^3^. Both neurons and glia cells are differentiated from neural stem/progenitor cells (NSCs/NPCs) during brain development, which processes are generally termed as neurogenesis and gliogenesis, respectively^1^,^4^. Gliogenesis starts later than neurogenesis during cortical development and it is stimulated by the newborn neurons^4^. However, the molecular mechanisms controlling the neurogenesis to gliogenesis switch are still not very clear; and deregulated or defective gliogenesis has been indicated in many neural developmental diseases such as autism and schizophrenia^1^. Several important signal pathways such as Notch and CT-1/JAK/STAT signaling pathways have been shown to be involved in gliogenesis^5^,^6^.

Notch signaling acts as an important and delicate molecular switch for NSCs cell fate during mammalian cortical development^5^,^6^. Four Notch receptors, NOTCH1-4, have been identified so far^7^. NOTCH1, the most studied one, is required for multiple cell fate determination processes, such as maintaining of NSCs pluripotency^1^,^8^, promoting astrocytes differentiation^9^,^10^,^11^ and neuronal maturation^12^,^13^,^14^. Interestingly, activated NOTCH1 signaling is essential for both NSC self-renewing and gliogenesis while down-regulation of NOTCH1 signaling is required for neurogenesis^5^,^15^,^16^,^17^. It is not clear how NOTCH1 signaling is switched between those processes, especially how it is up-regulated during gliogenesis^4^.

NOTCH1 pathway activation involves three proteolytic cleavage steps. First, NOTCH1 is processed by a Furin-like enzyme in the Golgi apparatus and the self-heterodimer NOTCH1 receptor is transported to cell membrane. After binding to the NOTCH1 ligands on neighboring cells, such as Jagged 1 (JAG-1) or Delta-like 1(DLL-1), NOTCH1 is cleaved by the site 2 (S2) enzymes, a disintergrin and metalloprotease 10 and/or 17 (ADAM10 & ADAM17), at outside juxtamembrane region^18^. Subsequently the transmembrane stub is cleaved by γ-secretase at site 3 (S3) to generate NOTCH1 intracellular domain (NICD). Within those proteolytic steps, the S2 cleavage is the rate-limiting step for NOTCH1 activation^19^. NICD translocates to the nucleus and interacts with transcription factor CSL (CBF1, Su(H), Lag1) to activate downstream targets such as Hes1, Hes5, and nuclear factor I/A (NFIA)^16^,^19^. It has been shown that NOTCH1 activates NFIA to release DNA (cytosine-5)-methyltransferase 1 (DNMT1) from the promoter of Glial fibrillary acidic protein (GFAP, a glia marker protein) during gliogenesis^5^,^9^,^20^,^21^. Interrupting NOTCH1 signaling pathway by deletion of *CBF-1* can cause severe defects in gliogenesis^22^.

In addition to Notch, cardiotrophin-1 (CT-1), an interleukin-6 (IL-6) family cytokine secreted by newborn neurons, is also involved in gliogenesis^1^,^23^,^24^,^25^,^26^. CT-1 stimulates gliogenesis by activating the JAK/STAT pathway and knockout of *CT-1* in mouse brain causes the reduction of glia cells^24^,^25^. Previous studies also showed that early activation of JAK/STAT pathway in NSCs leads to the premature generation of glial cells in embryonic mouse brain, while inhibition of the JAK/STAT pathway prevents the production of glia cells^27^. In addition, the expression of GFAP requires simultaneous activation of both NOTCH1 and JAK/STAT pathways as both CBF-1 and STAT3 bind to the promoter region of GFAP^9^. Therefore, both NOTCH1 and CT-1/JAK/STAT signals are essential for gliogenesis.

To ensure a synchronized activation of both NOTCH1 and CT-1/JAK/STAT pathways during gliogenesis, signal crosstalk between the two pathways has been proposed^16^. It has been shown that NOTCH1 activation leads to STAT3 activation^28^. On the other hand, STAT3 can induce NOTCH1 ligands expression^21^. To further understand the relationship between the NOTCH1 and CT-1 pathways during gliogenesis, we investigated the role of CT-1 on NOTCH1 activation. Here we show that CT-1 can activate NOTCH1 signaling through the induction of ADAM10 expression. Myc-associated zinc finger protein (MAZ) plays an essential role in CT-1 stimulated ADAM10 expression and gliogenesis. Our results revealed a novel mechanism for the regulation of NOTCH1 signaling by CT-1 which is essential for gliogenesis.

## Results

### CT-1 induces the levels of ADAM10 and NICD in NPCs

**Figure 1:**
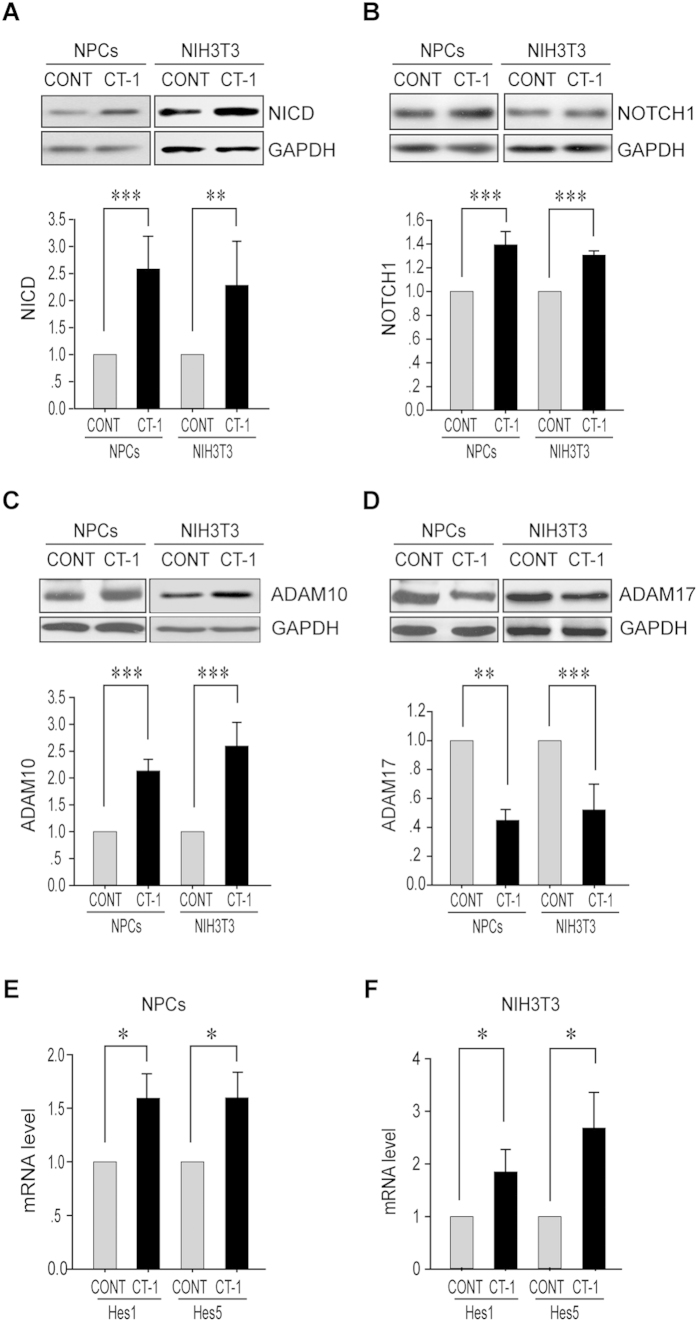
CT-1 up-regulates ADAM10 and NICD levels in NPCs and NIH3T3 cells. NPCs isolated from E11.5–13.5 mouse embryonic cortex were stimulated with CT-1 (100 ng/ml) or buffer (control, Cont) for 72 hrs, and NIH3T3 cells were stimulated with CT-1 (100 ng/ml) or buffer (control, Cont) for 24 hrs. Cell lysates were subjected to Western Blot analysis for both NICD **(A)** and full length NOTCH1 receptor **(B)** and two S2 enzymes ADAM10 **(C)** and ADAM17 **(D)**. GAPDH was used as loading control. Representative blots and statistical analysis are shown in the upper and lower panels respectively. Two downstream effectors of NOTCH1 pathway, *Hes1* & *Hes5* were examined by qRT-PCR after CT-1 stimulation for 24 hrs in NIH3T3 cells **(E)** or 48 hrs in NPCs **(F)**. The blots were cropped to improve the clarity and conciseness and full-length blots are presented in Supplementary Fig. S3. All data represent means ± SEM (one-way ANOVA). N ≥ 5, *p < 0.05, **p < 0.01, ***p < 0.001.

Since both Notch and CT-1 signal pathways are involved in gliogenesis, we firstly investigated whether CT-1 has any effect on NOTCH1 pathway. Cultured NPCs isolated from embryonic day 11.5–13.5 (E11.5–13.5) mouse frontal cortex were treated with CT-1 for 72 hrs. The levels of several important components in the NOTCH1 pathway including NOTCH1 ligands (JAG-1 and DLL-1), S2 enzymes (ADAM10 and ADAM17), full length NOTCH1 receptor and NICD were inspected. As shown in Fig. 1A, the level of NICD was increased ∼158% after CT-1 stimulation, while the full length NOTCH1 receptor was moderately enhanced ∼39% (Fig. 1B). On the other hand, JAG-1 and DLL-1 levels were apparently not affected (Fig. S1A,B). Interestingly, the level of ADAM10 was induced ∼112% by CT-1, which is comparable to that of NICD (Fig. 1C). However, the level of ADAM17 was decreased by ∼55% (Fig. 1D). Similar effects were also observed in the mouse embryonic fibroblast cell line NIH3T3 cells with 24 hrs CT-1 stimulation (Fig. 1A–D).

To confirm that the induction of ADAM10 and NICD by CT-1 leads to the NOTCH1 pathway activation, we examined two NOTCH1 down-stream targets, *Hes1* and *Hes5*. The mRNA levels of both *Hes1* and *Hes5* were significantly induced by CT-1 stimulation in both NPCs (Fig. 1E) and NIH3T3 cells (Fig. 1F). Thus, our results indicate that CT-1 stimulation can elevate both ADAM10 and NICD levels and activate NOTCH1 signaling.

### ADAM10 is essential for CT-1 induced NOTCH1 cleavage

**Figure 2:**
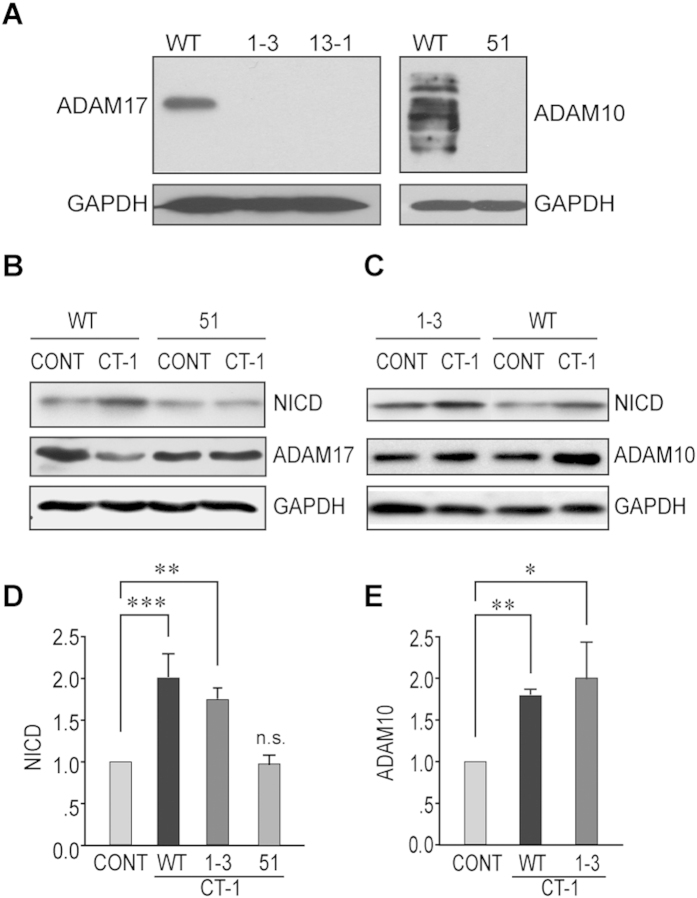
ADAM10 is essential for CT-1 induced NICD increase. **(A)** Confirmation of ADAM17 KO 293T cell lines (1–3 and 13–1) and ADAM10 KO 293T cell line (51) by Western blot analysis. **(B,C)** Different cell lines were stimulated with 100 ng/ml CT-1 for 24 hrs. Cell lysates were analyzed for NICD, ADAM10, or ADAM17 levels with GAPDH as loading control. Representative blots are shown in (**B,C**). **(D,E)** Statistical analysis for NICD and ADAM10 levels from (**B**,**C**). The blots were cropped to improve the clarity and conciseness and full-length blots are presented in Supplementary Fig. S3. All data represent means ± SEM (one-way ANOVA). N = 4, *p < 0.05, **p < 0.01, ***p < 0.001.

As CT-1 could induce ADAM10 but not ADAM17 and activate NOTCH1 signaling, we postulated that CT-1 might activate NOTCH1 signaling through ADAM10. To test this hypothesis, both ADAM10 and ADAM17 knockout cell lines were generated with TALEN method as described in *materials and methods*. The knockout of ADAM10 or ADAM17 was confirmed by Western blot analyses (Fig. 2A). Similar to NIH3T3 cells, the levels of NICD and ADAM10 were up-regulated after CT-1 stimulation for 24 hrs in WT cells (Fig. 2B–E). CT-1 was still able to up-regulate both ADAM10 and NICD levels in ADAM17 KO cells (Fig. 2C–E). However, the elevation of NICD level was not observed in ADAM10 KO cells (Fig. 2B,D), indicating that ADAM10 but not ADAM17 is essential for CT-1 stimulated NICD elevation.

### CT-1 induces the transcription of ADAM10 through MAZ

**Figure 3:**
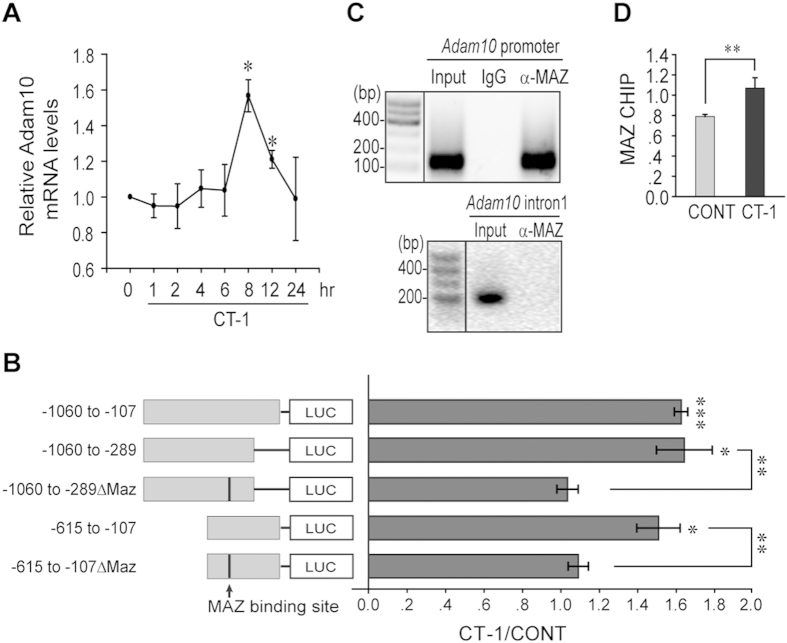
CT-1 induces ADAM10 transcription through MAZ. **(A)** NIH3T3 cells were treated with CT-1 at different time points. *Adam10* mRNA levels were determined by qRT-PCR. **(B)** Luciferase reporter constructs with different regions of *Adam10* promoter were transfected into NIH3T3 cells. Relative luciferase activities were determined after stimulation with CT-1 or control buffer for 12 hrs. **(C)** After incubation with CT-1 for 24 hrs, nuclei of NIH3T3 cells were extracted and ChIP were performed with anti-MAZ antibody or IgG control followed by amplification with primers targeting the −526 to −410 bp of *Adam10* promoter region. An adjacent fragment of *Adam10* intron1 was included as a negative control. ‘Input’ indicates PCR amplification of total DNA. **(D)** The relative amount of *Adam10* promoter fragments by CHIP assay in (**D**) were determined by qRT-PCR. All data represent means ± SEM (one-way ANOVA). N = 4, *p < 0.05, **p < 0.01, ***p < 0.001.

We went on to explore how CT-1 induces the expression of ADAM10 and found that *Adam10* mRNA level was induced at 6 hrs after CT-1 stimulation, with a peak at 8 hrs in NIH3T3 cells (Fig. 3A), suggesting that CT-1 regulates the transcription of ADAM10. We therefore tried to find the potential important element(s) in the promoter of *ADAM10*. Luciferase reporter constructs with different regions of mouse *Adam10* promoter were generated and used for transcriptional analysis. As shown in Fig. 3B, *Adam10* promoter regions from −1060 to −107 could significantly up-regulate the luciferase activity in the presence of CT-1, comparable to the induction of endogenous ADAM10 mRNA level (Fig. 3A). This suggests that a response element is located within this region.

Although STAT3 is the downstream transcription factor of CT-1 in the up-regulation of GFAP expression during gliogenesis^25^, there is no STAT3 binding motif located within the ADAM10 promoter region excluding STAT3 as the responsible transcriptional factor^29^. Therefore, we characterized *Adam10* promoter furthermore and found that the −615 to −107 and −1060 to −289 could also respond to CT-1 stimulation (Fig. 3C). These suggest that the promoter region around −615 to −289 is potentially important. Using two online bioinformatics tools (www.cbil.upenn.edu/tess and www.tools.genome.duke.edu/ generegulation/mcpromoter), we searched for potential protein binding motif in that region and found a Myc-associated zinc finger protein (MAZ) binding motif CCCTCCC located at −458 to −452. Therefore, two deletion mutants (−1060 ~ −289ΔMAZ & −615 ~ −107ΔMAZ) were generated and tested. The deletion of MAZ binding motif completely abolished their response to CT-1 stimulation (Fig. 3B). CHIP assays confirmed that MAZ could indeed bind to the *Adam10* promoter as the anti-MAZ antibody could pull down the fragment of *Adam10* promoter which contained the MAZ binding motif but not the *Adam10* intron1 (Fig. 3C). Furthermore, CHIP assay also showed that CT-1 up-regulated the binding of MAZ to *Adam10* promoter (Fig. 3D). Taken together, our results indicate that the transcription factor, MAZ, plays a role in CT-1 stimulated transcriptional regulation on *ADAM10*.

### MAZ is important for CT-1 induced gliogenesis

**Figure 4:**
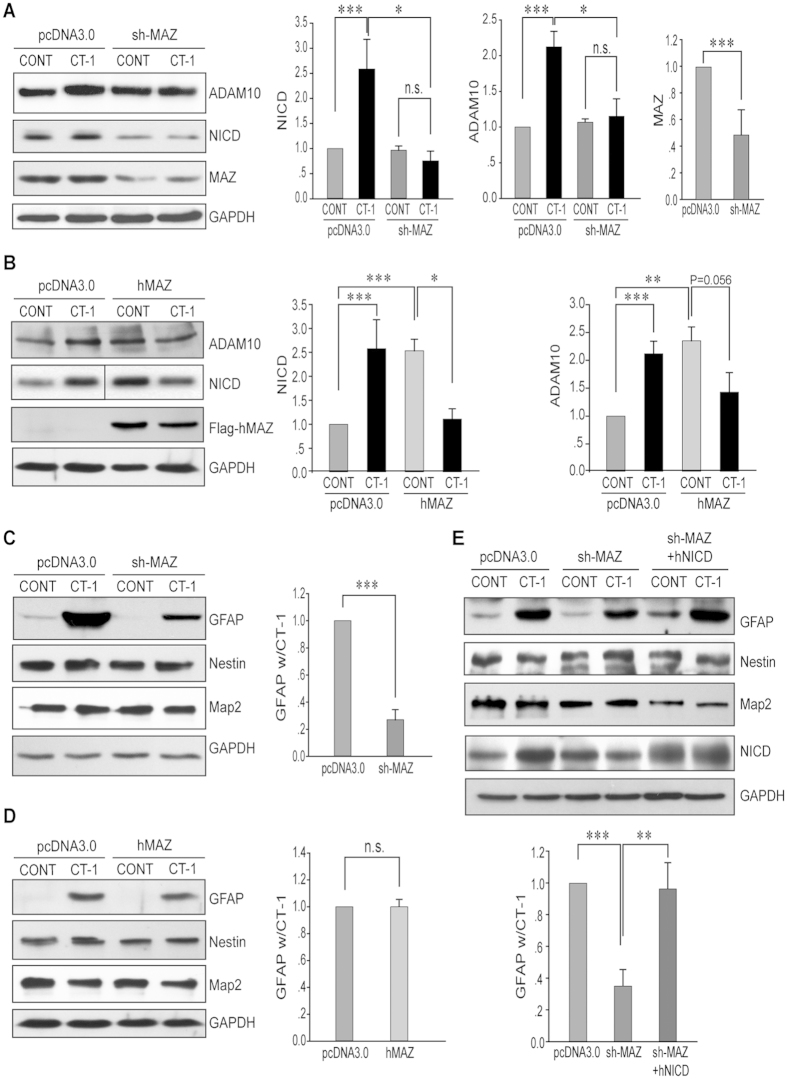
MAZ regulates CT-1 induced expression of NICD, ADAM10 and GFAP in NPCs. NPCs isolated from E11.5–13.5 mouse embryonic cortex were transfected with MAZ, sh-MAZ, hNICD or control constructs as indicated before seeding onto culture dishes. Cell lysates were prepared 72 hrs after CT-1 stimulation and NICD, ADAM10, MAZ, GFAP, Nestin or Map2 levels were analyzed by Western blot analysis. The blots were cropped to improve the clarity and conciseness and full-length blots are presented in Supplementary Fig. S3. All data represent means ± SEM (one-way ANOVA). N ≥ 4, *p < 0.05, **p < 0.01, ***p < 0.001.

We showed above that MAZ is responsible for CT-1 induced ADAM10 up-regulation, we then examined the role of MAZ in NPCs. NPCs were transfected with either MAZ-shRNAs or human MAZ (hMAZ) expressing construct and incubated with either CT-1 or control media. MAZ knockdown blocked CT-1 stimulated up-regulation of ADAM10 and NICD in NPCs (Figs 4A and S2). On the other hand, both ADAM10 and NICD levels were enhanced by hMAZ in the absence of CT-1 stimulation (Figs 4B and S2). When NPCs over-expressing hMAZ were stimulated with CT-1, the levels of NICD and ADAM10 were not further induced but down-regulated relatively (Figs 4C and S2).

**Figure 5:**
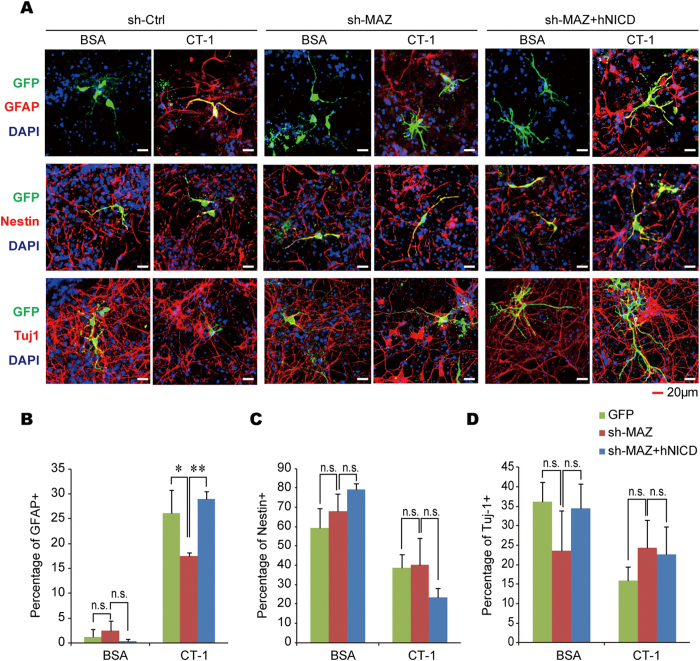
MAZ is required for CT-1 induced gliogenesis of NPCs. NPCs were isolated from E13.5–14.5 mouse embryonic cortex and transfected with GFP, sh-MAZ, hNICD or control constructs as indicated. Cells were treated with or without CT-1 for 72 hrs and subjected to immunofluorescent analysis. Nestin, Tuj1, and GFAP positive (^+^) cells were counted in those GFP positive cells. **(A)** The representative images. **(B–D)** Statistical analyses of the percentage of GFAP+, Nestin+, and Tuj-1+ cells in GFP+ cells. All data represent means ± SEM (one-way ANOVA). N ≥ 4, *p < 0.05, **p < 0.01, ***p < 0.001.

We then went on to investigate whether MAZ is involved in gliogenesis. Specific markers were adopted for the detection of cell lineages, with GFAP for astrocytes, Nestin for NPCs, and Map2 or Tuj1 for neurons. Similar to previous report^25^, CT-1 stimulation of NPCs substantially induced the expression of GFAP as well as the percentage of GFAP+ cells (Figs 4C and S2, Fig. 5A,B). Such effect could be significantly blocked by MAZ knock-down (Figs 4C and S2, Fig. 5A,B). Meanwhile, the levels of Nestin or Map2 or the ratio of Nestin+ or Tuj1+ cells were affected but not very significantly by MAZ knock-down (Figs 4C and 5C,D). We also inspected whether the MAZ expression could enhance the levels of GFAP. No apparent increase of GFAP expression was observed (Figs 4D and S2). Those results indicate that MAZ may play a necessary but not sufficient role in gliogenesis.

Since MAZ regulates the level of ADAM10 as well as NICD, we hypothesized that MAZ might act upstream of NOTCH1 cleavage in the NOTCH1 signaling pathway. To test this speculation, we co-expressed either mouse ADAM10 or human NICD fragment together with MAZ shRNAs in NPCs and inspected different cellular markers 72 hrs after CT-1 stimulation. The up-regulation of GFAP level under CT-1 stimulation was repressed by MAZ knockdown, and the down-regulation could be rescued by over-expression of either ADAM10 (Fig. S2) or hNICD (Fig. 4E). Similarly, the increased percentage of GFAP+ cells under CT-1 stimulation on NPCs was also repressed by MAZ knockdown and the suppression could be rescued by over-expression of hNICD (Fig. 5A,B). This indicates that the activation of NOTCH cleavage could rescue the inhibitory effects of MAZ knockdown on CT-1 induced gliogenesis. Taken together, our results strongly suggested that MAZ plays an important role in CT-1 induced gliogenesis of NPCs through the up-regulation of ADAM10 and NOTCH1 cleavage.

## Discussion

**Figure 6:**
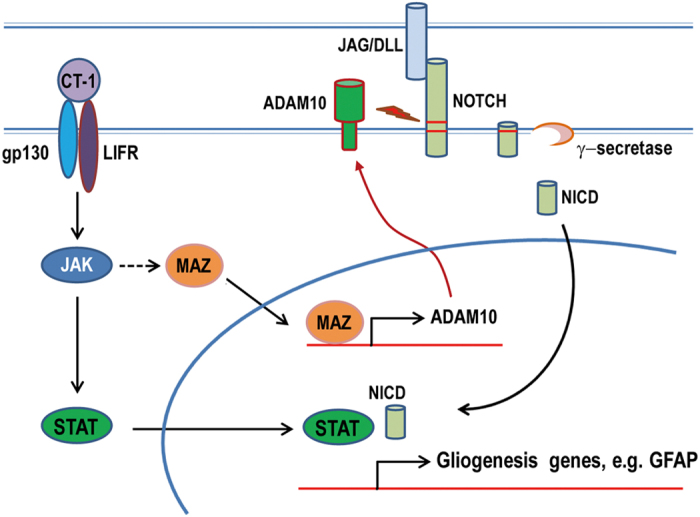
A schematic model for the crosstalk between CT-1 and NOTCH signalings during gliogenesis. CT-1 plays an important role in ensuring co-activation of JAK/STAT and Notch1 signaling during gliogenesis. CT-1 regulates the rate-limiting S2 enzymes of NOTCH, ADAM10 via transcription factor, Maz. Maz is essential for CT-1 induced up-regulation of ADAM10, NICD and GFAP, as well as gliogenesis.

Signal crosstalk between the NOTCH1 and CT-1/JAK/STAT pathways has been proposed to ensure a synchronized activation of both pathways for proper gliogenesis^16^. In this study, we demonstrate that CT-1 induces NOTCH1 signaling through the regulation of the speed-limiting S2 enzyme ADAM10. CT-1 can induce ADAM10 expression via transcription to ensure NOTCH1 activation and proper gliogenesis. MAZ is an essential transcriptional factor for CT-1 induced ADAM10 up-regulation and gliogenesis (Fig. 6). Our results revealed an interesting and important crosstalk mechanism between NOTCH1 and CT-1 signaling pathways for gliogenesis.

It has been shown previously that ciliary neurotrophic factor (CNTF) stimulation of embryonic stem cells^8^ or NSCs^30^ induces Notch activation as well as gliogenesis. Both CNTF and CT-1 belong to the IL-6 family cytokine, but CNTF is not expressed in embryonic brain while CT-1 is secreted by newborn neurons to stimulate gliogenesis of NSCs in the brain^25^. Unlike CNTF which promotes NOTCH1 receptor expression dramatically^31^, we showed that CT-1 only moderately enhances the level of NOTCH1 receptor. Instead, CT-1 induces ADAM10 level significantly.

ADAM10, the critical S2 enzyme of NOTCH1, has been considered as the physiological enzyme for NOTCH1 due to the similar phenotypes, including undeveloped heart and neural tube which lead to early embryonic lethality at E9.5, in *Adam10* and *Notch1* knockout mice^19^,^32^,^33^,^34^. Conditional ADAM10 knockout in mouse brain leads to disturbed cerebral cortex layout and less NSCs in cortex^35^. However, the role of ADAM10 in gliogenesis was not clear as the conditional knockout of ADAM10 was driven by Nestin, which starts to express early during NSCs proliferation stage. Recently we reported that ADAM10 co-localizes with NICD and partially with glial cell marker S100β in the intermediate zone (IZ) and cortical plate (CP) in E18.5 mouse cortex and ADAM10 expression in the IZ and CP is much higher than that in stem layer VZ and SVZ^36^. Considering that both NICD and ADAM10 were stimulated by CT-1, it is possible that higher levels of ADAM10 and NICD in the IZ and CP are likely to be stimulated by CT-1 secreted by surrounding newborn neurons.

In this study, we find that MAZ acts as an important transcription factor for the up-regulation of ADAM10 and NICD in response to CT-1 stimulation. In NPCs, CT-1 stimulation induced gliogenesis can be blocked by MAZ knockdown and this inhibitory effect can be rescued by both ADAM10 and NICD over-expression. Previous studies on MAZ, also named SAF-1 or Pur-1, were focused on its function as a downstream transcription factor of IL-1 or IL-6 signal during inflammation^37^. Very little is known for its function during development. Interestingly, it is reported recently that when the binding of MAZ was blocked with a *cis* double-stranded oligodeoxynucleotides in a neural stem cell line C17.2 cells, the cells tend to differentiate into neurons^38^. This is consistent with our results that MAZ knockdown leads to suppression of NOTCH signaling. Because down-regulation of NOTCH signaling is required for neurogenesis^5^,^15^,^16^,^17^, inhibition of MAZ function in C17.2 cells may attenuate NOTCH signaling and promotes neurogenesis. Further understanding will be needed in conditional knockout mice model on MAZ and/or CT-1.

An interesting observation is that over-expression of hMAZ alone without CT-1 stimulation did not induce the level of GFAP, even though the level of NICD was induced in NPCs. This suggests that MAZ plays a necessary but not sufficient role in gliogenesis. MAZ may have to coordinate with other components in CT-1 signaling pathways, e.g. JAK/STAT, to induce gliogenesis.

Another interesting observation is that the level of total MAZ was decreased about 43% after CT-1 stimulation for 72 hrs in NPCs (Figs S2A and E), even though CT-1 could up-regulate the binding of MAZ to ADAM10 promoter (Fig. 3D). This suggests that a negative feedback signal may exist here in the regulation of MAZ expression. However, the levels of ADAM10 and NICD are down-regulated instead of further induced by CT-1 in MAZ over-expressing NPCs cells (Figs 4B and S2A,C,D). Therefore, it is very likely that CT1 stimulation in the presence of high level of MAZ may also activate negative feedback signal to prevent the further elevation of ADAM10 and NICD levels. Our results suggest that the components involved in gliogenesis are well coordinated. How and why such sophisticated mechanism is required for gliogenesis will be an interesting subject for future study.

In summary, the gliogenesis of NSCs/NPCs requires both CT-1 and NOTCH1 signal pathways. In addition to activation of the JAK/STAT pathway, CT-1 also up-regulates Notch signaling through the regulation of S2 enzyme ADAM10. Such collaborative and synergic crosstalk between the two essential pathways is important to ensure the proper gliogenesis during brain development.

## Material and Methods

### Antibodies and inhibitors

The following antibodies were purchased from Abcam: ADAM10 (ab1997), NICD (ab8925), GFAP (ab7260), NOTCH1 (ab27526), DLL-1 (ab76655), JAG-1 (ab7771); and Nestin (ab6142), MAP2 (ab11268). Goat anti-ADAM17 (sc6416) and rabbit anti-MAZ (sc-28745) antibodies were purchased from Santa Cruz Biotech. Mouse anti-GAPDH, anti-alpha Tubulin, anti-beta Actin, and goat anti-rabbit and anti-mouse secondary antibodies were all purchased from Kangchen Bio-tech. Goat anti-Rat secondary antibody was purchased from Beyotime. CT-1 peptide (300–32) was purchased from Peprotech.

### Plasmids

pCMV6-flag-hMAZ construct was purchased from Origene. Human NICD construct was a gift from Dr. Hongyan Wang. Mouse MAZ shRNA constructs were purchased from Genechem Inc. Three different hairpins were cloned into pGV102/GFP to generate MAZ-shRNA constructs: 5′-gaTGCTGAGCTCGGCTTATATctcgagATATAA GCCGAGCTCAGCATC-3′, 5′-ggCCCTTCAAATGTGAGAAATctcgagATTTCTCACATTT GAAGGGCC-3′, 5′-gaGTAAGGTTGGGTGGTTAAActcgagTTTAACCACCCAACCTTA CTC-3′, named as MAZ-shRNA1, 2, 3, respectively. Scramble sequence for control shRNA was: 5′-CACCGTTCTCCGAACGTGTCACGTCAAGAGATTACGTGACAC GTTCGGAGAATTTTTTG-3′.

To generate ADAM10 luciferase reporter constructs, different regions of the mouse *Adam10* promoter (NM_11487) were generated by PCR and cloned into NheI/HindIII sites of pGL3. The primers used are listed below. −1060 F: 5′-ctagctagcgccccgctcctctcctc-3′; −615 F: 5′-ctagctagcttttgga ggcgaagaagc-3′; −451 F: 5′-aggccaatccctgctctccg-3′; −459 R: 5′-cggggccgctggagactccg-3′; −289 R: 5′-cccaagcttcgcgac ggcacccaatac-3′; −107 R: 5′-cccaagcttgaagcgcctccctctcg-3′; −106 R: 5′-cccaagcttcgaagcgcctccctctc-3′. The −615 to −107 ΔMAZ (deleted −458 to −452) construct was generated by two fragments and vector ligation directly. The −1060 to −289 ΔMAZ (deleted −458 to −452) construct was generated with NovoRec® PCR one-step cloning kit (Sino Biotech) using NovoRec recombinase. Two circles of PCR and recombination reactions were used to generate the deletion construct. The primers used were −1060 rec-F1: 5′-agaacatttctctatcgataGCCCCGCTCCTCTCCTCCCA-3′ and −459 rec-R1: 5′-gatctcgagcccgggctagcCGGGGCCGCTGGAGACTCCG-3′; −451 rec-F2: 5′-cggagtctccagcggccccgAGGCCAATCCCTGCTCTCCG-3′ and −289 rec-R2: 5′-accaacagtaccggaatgccCGCGACGGCACCCAATAC-3′. Small letters indicate the recombinant arms used in the reactions.

### Cell culture, transfection & Western Blot

NIH3T3 and 293T cells were grown in DMEM supplemented with 10% fetal bovine serum, 2 mmol/L glutamine, and 1% penicillin/streptomycin. Cells were transfected using Fugene (Roche 14738300) or X-tremeGENE 9 (Roche 06365787001). CT-1 was dissolved in DMEM containing 0.1% BSA. The following stimulator and inhibitors were used: 100 ng/ml CT-1, and 10 μg/ml Cycloheximide. After proper incubation time, the cells were washed with PBS, and then lysed in cell lysis buffer (PBS, pH 7.4, with 1% (v/v) TritonX-100, 1× protease inhibitors, and 5 mmol/L 1,10- phenanthroline). Lysates were subjected to Western blot. In brief, samples were loaded and separated on SDS-PAGE and then electrically blotted to a polyvinylidene difluoride (PVDF) membrane (Bio-Rad Laboratories). After incubation in blocking buffer for 1 h, the membrane was incubated with the primary antibody at 4 °C overnight and then with the HRP-conjugated secondary antibody for 1 h, ECL was used to visualizing the protein bands. All films were scanned and analyzed with Quantity ONE based on intensity and normalized to GAPDH.

### TALEN knockout 293T cell line

ADAM17 and ADAM10 knockout 293T cell line was made with TALEN technique by Viewsolid Biotech. In brief, TALEN primers were designed to target exon 1 of *ADAM17* (NM_003183.4): 5′-CTCAGACTACGATATTCTctcb tttatctaatATCCAGCAGCATTCGGT-3′ or the exon 8 of *ADAM10* (NM_001110.2): 5′-TTTTGATGATGGCGTACttggtctggcttgggTTGGAGCACCTTCAGGTA-3′. Capital letters are the reading frame for TALEN arms. Two *ADAM17* KO cell lines, 1–3 and 13–1 and one ADAM10 KO cell line 51 were successfully generated. A frame shift was generated after 138bp of human *ADAM17* CDS and a stop cordon was generated at 168bp or 159bp for 1–3 or 13–1, respectively. For ADAM10 knockout cell line 51, a frame shift after 990bp of *ADAM10* CDS and an early stop was generated at 1026bp.

### NPCs isolation and differentiation assay

NPCs was isolated from C57BL/6 or CD-1(ICR) mice purchased from Shanghai SLAC laboratory animal Inc or Institute of Genetics and Developmental Biology (IGDB), Chinese Academy of Sciences (CAS). This study was carried out in strict accordance with the recommendations in the Guide for the Care and Use of Laboratory Animals of Fudan University and IGDB, CAS. The protocol was approved by the Committee on the Ethics of Animal Experiments of Fudan University, Shanghai, and IGDB, CAS, Beijing. NPCs isolation and culture were based on previous report^39^. In brief, E11.5–14.5 embryonic frontal cortex were dissected in cold PBS and NPCs were cultured in Neurobasal medium (Invitrogen) containing 500 μM glutamine, 2% B27 supplement (Sigma), 1% penicillin and streptomycin (Invitrogen) and 40 ng/ml FGF2 (Peprotech). Transfections on NPCs were performed with X-tremeGENE HP DNA transfection reagent (Roche, 06366236001) before seeding the isolated NPCs on culture dishes. For CT-1 stimulated differentiation, NPCs were treated with CT-1 (100 ng/ml) for indicated time.

### qRT-PCR

NPCs and NIH3T3 cells were lysed and total RNA was extracted with RNAeasy kit (QIAGEN). The levels of ADAM10 mRNA were determined with qRT-PCR kit (Takara RR036A and RR820A)*. GAPDH* gene was used as internal control. The primers used were: *Adam10*:5′-cctgccatttcactctgtcattta-3′ & 5′-gtgcccgggctccttcctctactc-3′; *GAPDH*: 5′-acagcaactcccactcttccacct-3′ and 5′-ttgctcagtgtccttgctgggg-3′. *Hes1*, *Hes5* and *β-actin* primers were adopted from a previous report^40^.

### Luciferase reporter assay

NIH3T3 cells were transfected with 1 μg pGL3 with ADAM10 promoter controlled luciferase gene and 1 μg pSV-β-galactosidase using 4 μl Fugene or X-tremeGENE 9 in Opti-MEM (Invitrogen). After 12 hrs CT-1 stimulation, the media were changed and cells were lysed for measurement of luciferase activity using a luciferase assay system (Promega E1483) and galactosidase activity with β-galactosidase Reporter gene assay kit (Beyotime RG0036). Luciferase activity normalized to β-galactosidase activity represents the promoter activity.

### Chromatin Immunoprecipitation (CHIP) assay

ChIP with MAZ antibody was performed with Cell signaling SimpleCHIP® kit according to manufactory’s protocol. The enrichment of specific DNA sequences was determined by PCR with the following primers: 5′-aggtagcactttcacaggga-3′ and 5′-gccgctgcttcctgtccgct-3′. The PCR product is 117bp fragment correspondent to the −526 to −410 region of *Adam10* promoter. An irrelevant 204 bp fragment located on *Adam10* intron1 +1398 to +1601 was amplified with primers 5′-gtcctggctggctgttttcacttt-3′ and 5′-ctcttcacccacaatgcttatgct-3′.

### Immunofluorescent experiments

Cultured NPCs were washed with PBS and fixed in 4%PFA in PBS for 30 min at 4 °C. Then cells were treated with blocking buffer at room temperature (RT) for 1 hr. Primary antibody were added on cells and incubated at 4 °C overnight. Cells were washed with PBS 3 times next day before adding secondary antibody and incubated at RT for 1 hr. Cells were washed again with PBS and sealed for microscope imaging with Zeiss LSM700.

### Statistic analysis

All WB bands were quantified by using Quantity one software. The results were normalized to its corresponding loading control GAPDH. Then the CT-1 stimulated condition was compared to BSA treated control condition in each set of experiments. All experiments were repeated at least three times and the statistical significance was evaluated by one-way ANOVA test. Differences were taken as statistically significant at *p* < 0.05.

## Additional Information

**How to cite this article**: Liu, B. *et al.* MAZ mediates the cross-talk between CT-1 and NOTCH1 signaling during gliogenesis. *Sci. Rep.* **6**, 21534; doi: 10.1038/srep21534 (2016).

## Supplementary Material

Supplementary Information

## References

[b1] Freeman M. R. Specification and morphogenesis of astrocytes. Science 330, 774–778, 10.1126/science.1190928 (2010). 21051628 PMC5201129

[b2] Ullian E. M., Sapperstein S. K., Christopherson K. S. & Barres B. A. Control of synapse number by glia. Science 291, 657–661, 10.1126/science.291.5504.657 (2001). 11158678

[b3] Eroglu C. & Barres B. A. Regulation of synaptic connectivity by glia. Nature 468, 223–231, 10.1038/nature09612 (2010). 21068831 PMC4431554

[b4] Kriegstein A. & Alvarez-Buylla A. The glial nature of embryonic and adult neural stem cells. Annu Rev Neurosci 32, 149–184, 10.1146/annurev.neuro.051508.135600 (2009). 19555289 PMC3086722

[b5] Zhou Z. D., Kumari U., Xiao Z. C. & Tan E. K. Notch as a molecular switch in neural stem cells. IUBMB Life 62, 618–623, 10.1002/iub.362 (2010). 20681026

[b6] Yoon K. & Gaiano N. Notch signaling in the mammalian central nervous system: insights from mouse mutants. Nat Neurosci 8, 709–715, 10.1038/nn1475 (2005). 15917835

[b7] Bolos V., Grego-Bessa J. & de la Pompa J. L. Notch signaling in development and cancer. Endocr Rev 28, 339–363, 10.1210/er.2006-0046 (2007). 17409286

[b8] Ramasamy S. K. & Lenka N. Notch exhibits ligand bias and maneuvers stage-specific steering of neural differentiation in embryonic stem cells. Mol Cell Biol 30, 1946–1957, 10.1128/MCB.01419-09 (2010). 20154142 PMC2849467

[b9] Ge W. *et al.* Notch signaling promotes astrogliogenesis via direct CSL-mediated glial gene activation. J Neurosci Res 69, 848–860, 10.1002/jnr.10364 (2002). 12205678

[b10] Morrison S. J. *et al.* Transient Notch activation initiates an irreversible switch from neurogenesis to gliogenesis by neural crest stem cells. Cell 101, 499–510, 10.1016/S0092-8674(00)80860-0 (2000). 10850492

[b11] Namihira M. *et al.* Committed neuronal precursors confer astrocytic potential on residual neural precursor cells. Dev Cell 16, 245–255, 10.1016/j.devcel.2008.12.014 (2009). 19217426

[b12] Genoud S. *et al.* Notch1 control of oligodendrocyte differentiation in the spinal cord. J Cell Biol 158, 709–718, 10.1083/jcb.200202002 (2002). 12186854 PMC2174019

[b13] Givogri M. I., Schonmann V., Cole R., De Vellis J. & Bongarzone E. R. Notch1 and Numb genes are inversely expressed as oligodendrocytes differentiate. Dev Neurosci 25, 50–64, 10.1159/000071468 (2003). 12876431

[b14] Zhang Y. *et al.* Notch1 signaling plays a role in regulating precursor differentiation during CNS remyelination. Proc Natl Acad Sci USA 106, 19162–19167, 10.1073/pnas.0902834106 (2009). 19855010 PMC2776461

[b15] Hatakeyama J. & Kageyama R. Notch1 expression is spatiotemporally correlated with neurogenesis and negatively regulated by Notch1-independent Hes genes in the developing nervous system. Cereb Cortex 16 Suppl 1, i132–137, 10.1093/cercor/bhj166 (2006). 16766699

[b16] Pierfelice T., Alberi L. & Gaiano N. Notch in the vertebrate nervous system: an old dog with new tricks. Neuron 69, 840–855, 10.1016/j.neuron.2011.02.031 (2011). 21382546

[b17] Kaltezioti V. *et al.* Prox1 regulates the notch1-mediated inhibition of neurogenesis. PLoS Biol 8, e1000565, 10.1371/journal.pbio.1000565 (2010). 21203589 PMC3006385

[b18] Bray S. J. Notch signalling: a simple pathway becomes complex. Nat Rev Mol Cell Biol 7, 678–689, 10.1038/nrm2009 (2006). 16921404

[b19] van Tetering G. *et al.* Metalloprotease ADAM10 Is Required for Notch1 Site 2 Cleavage. The Journal of biological chemistry 284, 31018–31027, 10.1074/jbc.M109.006775 (2009). 19726682 PMC2781502

[b20] Anthony T. E., Mason H. A., Gridley T., Fishell G. & Heintz N. Brain lipid-binding protein is a direct target of Notch signaling in radial glial cells. Genes Dev 19, 1028–1033, 10.1101/gad.1302105 (2005). 15879553 PMC1091737

[b21] Kanski R., van Strien M. E., van Tijn P. & Hol E. M. A star is born: new insights into the mechanism of astrogenesis. Cell Mol Life Sci 71, 433–447, 10.1007/s00018-013-1435-9 (2014). 23907612 PMC11113452

[b22] Taylor M. K., Yeager K. & Morrison S. J. Physiological Notch signaling promotes gliogenesis in the developing peripheral and central nervous systems. Development 134, 2435–2447, 10.1242/dev.005520 (2007). 17537790 PMC2653864

[b23] Yoshimatsu T. *et al.* Non-cell-autonomous action of STAT3 in maintenance of neural precursor cells in the mouse neocortex. Development 133, 2553–2563, 10.1242/dev.02419 (2006). 16728475

[b24] Miller F. D. & Gauthier A. S. Timing is everything: making neurons versus glia in the developing cortex. Neuron 54, 357–369, 10.1016/j.neuron.2007.04.019 (2007). 17481390

[b25] Barnabe-Heider F. *et al.* Evidence that embryonic neurons regulate the onset of cortical gliogenesis via cardiotrophin-1. Neuron 48, 253–265, 10.1016/j.neuron.2005.08.037 (2005). 16242406

[b26] Bonni A. *et al.* Regulation of gliogenesis in the central nervous system by the JAK-STAT signaling pathway. Science 278, 477–483 (1997). 9334309 10.1126/science.278.5337.477

[b27] He F. *et al.* A positive autoregulatory loop of Jak-STAT signaling controls the onset of astrogliogenesis. Nat Neurosci 8, 616–625, 10.1038/nn1440 (2005). 15852015 PMC4222251

[b28] Kamakura S. *et al.* Hes binding to STAT3 mediates crosstalk between Notch and JAK-STAT signalling. Nat Cell Biol 6, 547–554, 10.1038/ncb1138 (2004). 15156153

[b29] Prinzen C., Muller U., Endres K., Fahrenholz F. & Postina R. Genomic structure and functional characterization of the human ADAM10 promoter. FASEB J 19, 1522–1524, 10.1096/fj.04-3619fje (2005). 15972296

[b30] Nagao M., Sugimori M. & Nakafuku M. Cross talk between notch and growth factor/cytokine signaling pathways in neural stem cells. Mol Cell Biol 27, 3982–3994, 10.1128/MCB.00170-07 (2007). 17371842 PMC1900030

[b31] Chojnacki A., Shimazaki T., Gregg C., Weinmaster G. & Weiss S. Glycoprotein 130 signaling regulates Notch1 expression and activation in the self-renewal of mammalian forebrain neural stem cells. J Neurosci 23, 1730–1741, 23/5/1730 (2003). 12629177 10.1523/JNEUROSCI.23-05-01730.2003 PMC6741977

[b32] Hartmann D. *et al.* The disintegrin/metalloprotease ADAM 10 is essential for Notch signalling but not for alpha-secretase activity in fibroblasts. Hum Mol Genet 11, 2615–2624, 10.1093/hmg/11.21.2615 (2002). 12354787

[b33] Gordon W. R. *et al.* Structure of the Notch1-negative regulatory region: implications for normal activation and pathogenic signaling in T-ALL. Blood 113, 4381–4390, 10.1182/blood-2008-08-174748 (2009). 19075186 PMC2676092

[b34] Gordon W. R. *et al.* Effects of S1 cleavage on the structure, surface export, and signaling activity of human Notch1 and Notch2. PLoS One 4, e6613, 10.1371/journal.pone.0006613 (2009). 19701457 PMC2726630

[b35] Jorissen E. *et al.* The disintegrin/metalloproteinase ADAM10 is essential for the establishment of the brain cortex. J Neurosci 30, 4833–4844, 10.1523/JNEUROSCI.5221-09.2010 (2010). 20371803 PMC2921981

[b36] Ma Z., Li Q., Zhang Z. & Zheng Y. A Disintegrin and Metalloprotease 10 in neuronal maturation and gliogenesis during cortex development. Neural Regen Res 8, 24–30, 10.3969/j.issn.1673-5374.2013.01.003 (2013). 25206368 PMC4107504

[b37] Ray A., Yu G. Y. & Ray B. K. Cytokine-responsive induction of SAF-1 activity is mediated by a mitogen-activated protein kinase signaling pathway. Mol Cell Biol 22, 1027–1035 (2002). 11809795 10.1128/MCB.22.4.1027-1035.2002 PMC134650

[b38] Wang J. *et al.* Regulation of neural stem cell differentiation by transcription factors HNF4-1 and MAZ-1. Mol Neurobiol 47, 228–240, 10.1007/s12035-012-8335-0 (2013). 22944911

[b39] Blanchot-Jossic F. *et al.* Up-regulated expression of ADAM17 in human colon carcinoma: co-expression with EGFR in neoplastic and endothelial cells. J Pathol 207, 156–163, 10.1002/path.1814 (2005). 16041691

[b40] Liu Q. *et al.* Co-culturing improves the OGD-injured neuron repairing and NSCs differentiation via Notch pathway activation. Neurosci Lett 559, 1–6, 10.1016/j.neulet.2013.11.027 (2014). 24284009

