# Corrigendum: MAZ mediates the cross-talk between CT-1 and NOTCH1 signaling during gliogenesis

**DOI:** 10.1038/srep23309

**Published:** 2016-03-29

**Authors:** Bin Liu, Anyun Ma, Feng Zhang, Yumeng Wang, Zengmin Li, Qingyu Li, Zhiheng Xu, Yufang Zheng

Scientific Reports 6: Article number: 21534;10.1038/srep21534 published online: 02122016; updated: 03292016

In this Article, the legend of Figure 1 contains a typographical error.

“Two downstream effectors of NOTCH1 pathway, *Hes1* & *Hes5* were examined by qRT-PCR after CT-1 stimulation for 24 hrs in NIH3T3 cells **(E)** or 48 hrs in NPCs **(F)**.”

should read:

“Two downstream effectors of NOTCH1 pathway, *Hes1* & *Hes5* were examined by qRT-PCR after CT-1 stimulation for 24 hrs in NIH3T3 cells **(F)** or 48 hrs in NPCs **(E)**.”

