# Supplementary material for: MAZ mediates the cross-talk between CT-1 and NOTCH1 signaling during gliogenesis
Source: Sci Rep. 2016 Feb 12;6:21534. doi: 10.1038/srep21534 (PMC4751466; doi:10.1038/srep21534)
Supplement: Supplementary Information [file srep21534-s1.pdf]

MAZ mediates the cross-talk between CT-1 and NOTCH1 signaling during gliogenesis

Bin Liu[1], Anyun Ma[1,2], Feng Zhang[3], Yumeng Wang[1,2], Zengmin Li[1,2], Qingyu Li[1], Zhiheng Xu[3,4], Yufang Zheng[1,2,4]

From [1]State Key Laboratory of Genetic Engineering and Ministry of Education (MOE) Key Laboratory of Contemporary Anthropology, School of Life Sciences, Fudan University, [2]The Institute of Developmental Biology and Molecular Medicine, Fudan University, Shanghai, China, 200433. [3]State Key Laboratory of Molecular Developmental Biology, Institute of Genetics and Developmental Biology, Chinese Academy of Sciences, Beijing, China 100101. [4]Innovation Center for International Cooperation of Genetics and Development, Fudan University, Shanghai, China, 200433.

Corresponding author: Dr. Yufang Zheng, School of Life Sciences, Fudan University, 2005 Songhu Road, Shanghai, China, 200438. Tel: 86-21-51630610. E-mail: zhengyf@fudan.edu.cn

Running title: CT-1 induces ADAM10 via MAZ

Keywords: CT-1; MAZ; ADAM10; NOTCH1; Gliogenesis

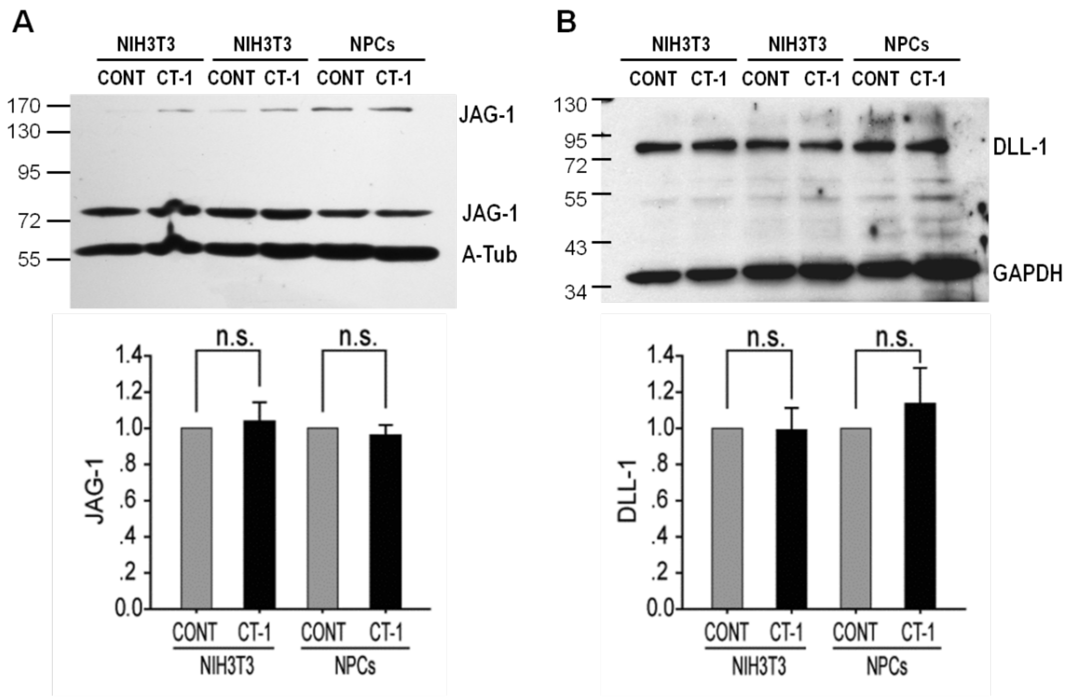

Figure S1. CT-1 has no effects on NOTCH1 ligands JAG-1 and DLL-1. NPCs isolated from E11.5-13.5 mouse embryonic cortex were stimulated with CT-1 (100ng/ml) or buffer (control, Cont) for 72hrs, and NIH3T3 cells were stimulated with CT-1 (100ng/ml) or buffer (control, Cont) for 24hrs. Cell lysates were subjected to Western Blot analysis for two NOTCH1 ligands JAG-1 **(A)** and DLL-1 **(B)**. GAPDH or alpha-Tubulin (A-Tub) were used as loading control. Representative blots and statistical analysis are shown in the upper and lower panels respectively. All data represent means ± SEM (one-way ANOVA). N⩾5, n.s. for *p*>0.05.

A

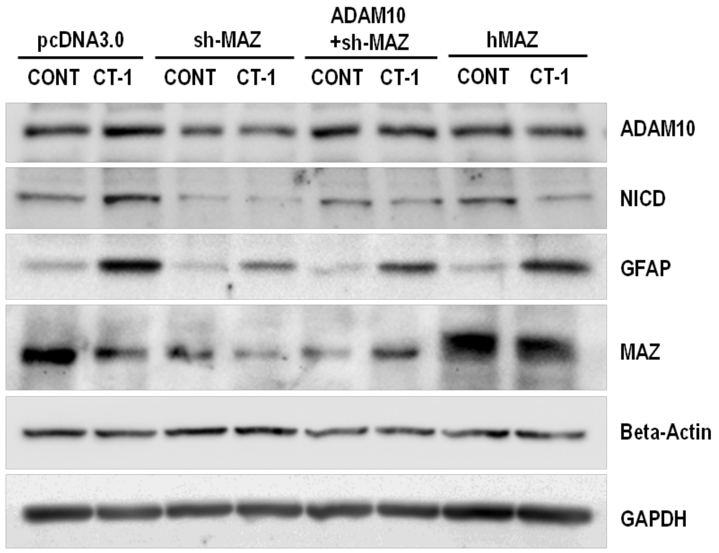

pcDNA3.0
sh-MAZ
ADAM10 +sh-MAZ
hMAZ
CONT CT-1 CONT CT-1 CONT CT-1 CONT CT-1
ADAM10
NICD
GFAP
MAZ
Beta-Actin
GAPDH

B

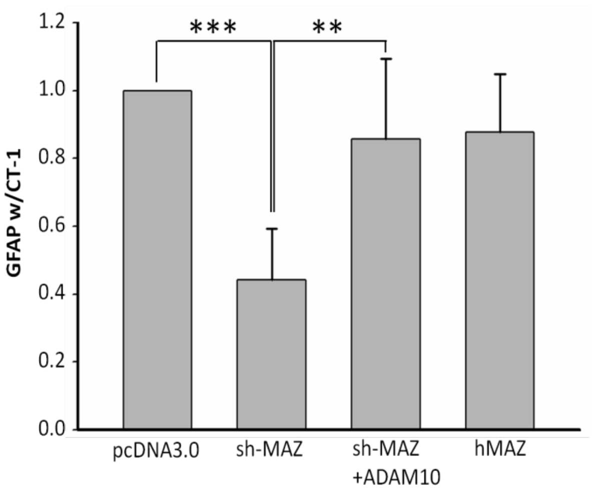

GFAP w/CT-1
1.2
1.0
0.8
0.6
0.4
0.2
0.0
***
**
pcDNA3.0
sh-MAZ
sh-MAZ +ADAM10
hMAZ

C

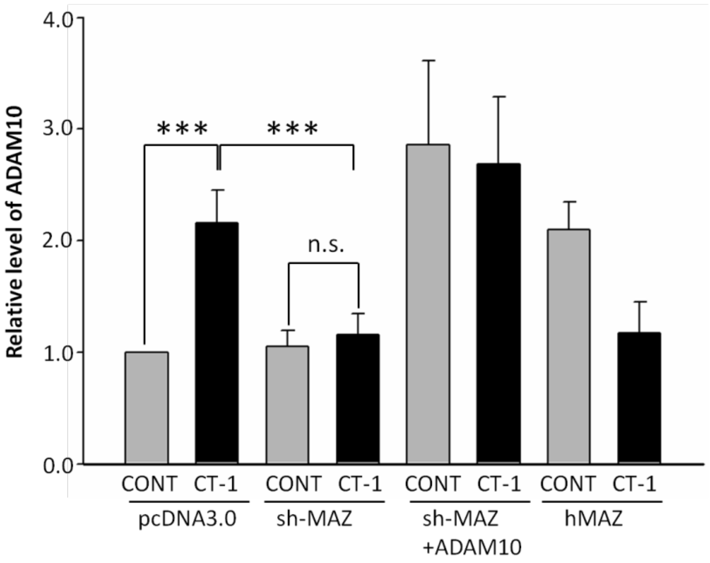

Relative level of ADAM10
4.0
3.0
2.0
1.0
0.0
***
***
n.s.
CONT CT-1 CONT CT-1 CONT CT-1 CONT CT-1
pcDNA3.0
sh-MAZ
sh-MAZ +ADAM10
hMAZ

D

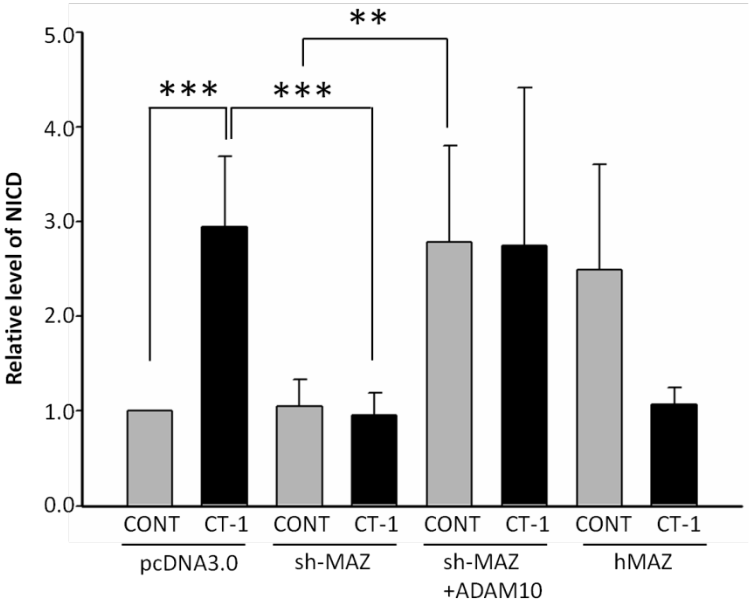

E

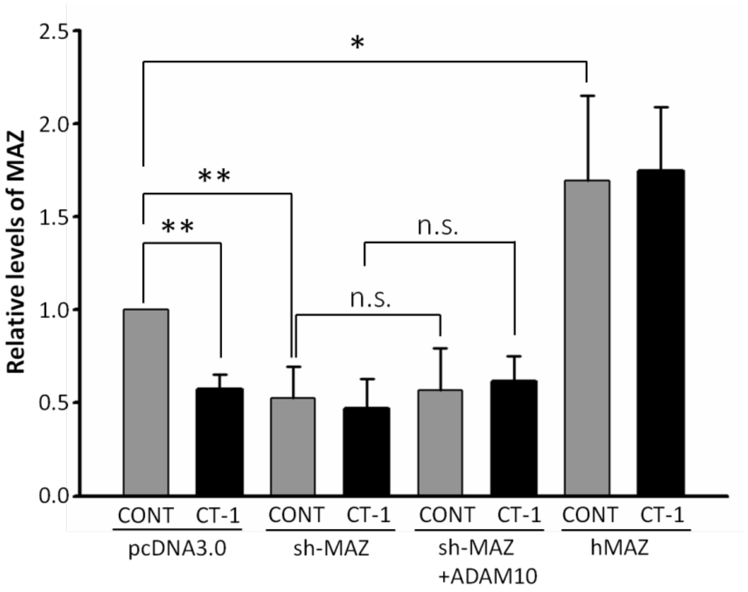

Figure S2. Over-expressing ADAM10 could rescue the effect of MAZ knock down on NPCs gliogenesis. NPCs isolated from E13.5 mouse embryonic cortex were transfected with designed plamids and then treated with CT-1 (100ng/ml) or buffer (CONT) for 72hrs. Cell lysates were subjected to WB analysis for GFAP, ADAM10, NICD, and MAZ. Beta-Actin and GAPDH were used as loading control. Representative blots are shown in **(A)** and statistical analysis are shown in **(B)** for GFAP, **(C)** for ADAM10, **(D)** for NICD, and **(E)** for MAZ respectively. All data represent means ± SEM (one-way ANOVA). N⩾4, n.s. for $p>0.05$. * $p<0.05$, ** $p<0.01$, *** $p<0.001$.

Figure S3. Full-length blots used in this study. NPCs or NIH3T3 cells were stimulated with CT-1 (100ng/ml) or buffer (control, Cont) as described before. In some cases, NPCs were transfected with designed plasmids and then treated with CT-1 for 72 hrs. Cell lysates were subjected to Western Blot analysis and GAPDH or alpha-Tubulin (a-Tub) were used as loading control. Representative blots were shown as below. The main bends on the blots were cropped and used in the main figures 1,2,and 4.

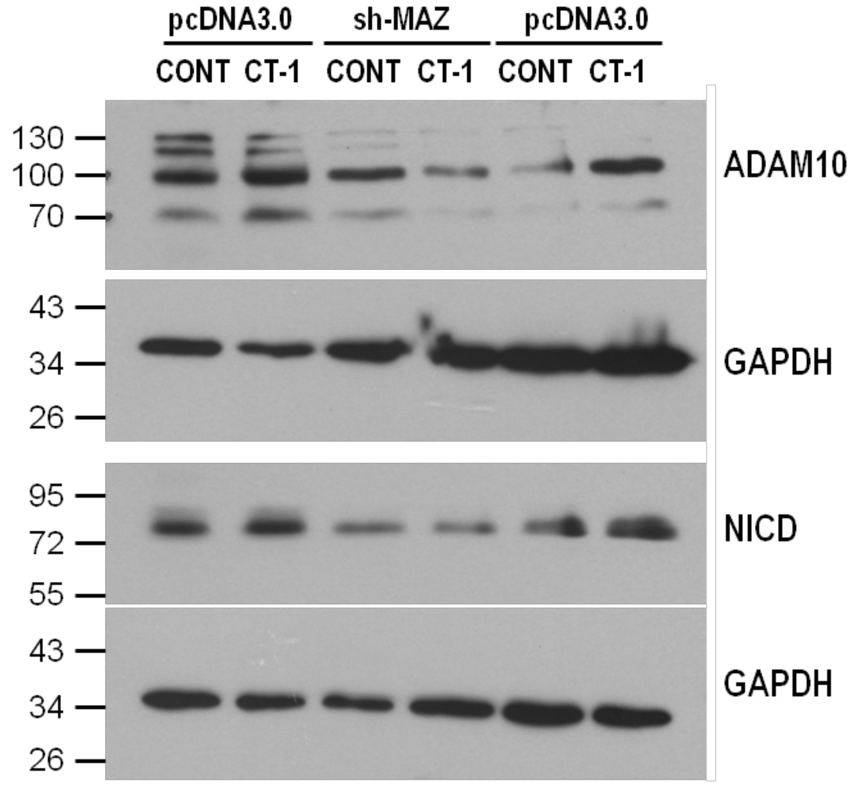

NICD and ADAM10 in MAZ knockdown NPCs for Fig.1A, 1C and 4A.

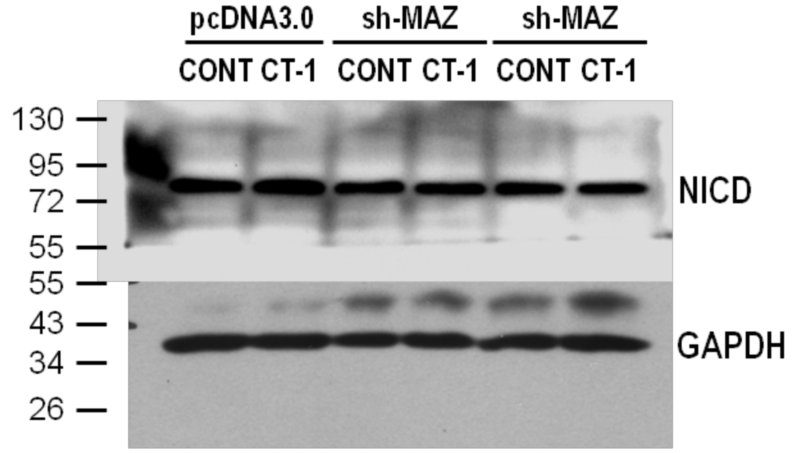

NICD expression in NIH3T3 cells for Fig.1A.

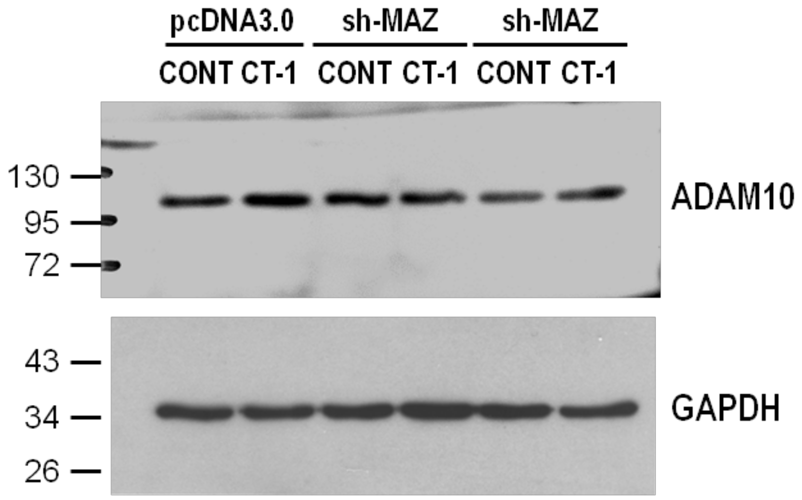

ADAM10 expression in NIH3T3 cells for Fig.1C.

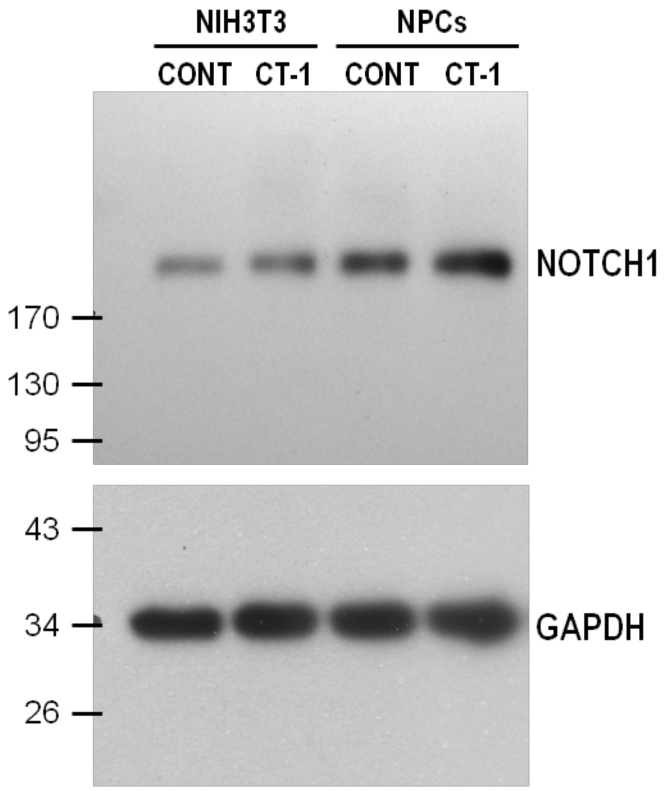

NOTCH1 expression for Fig.1B

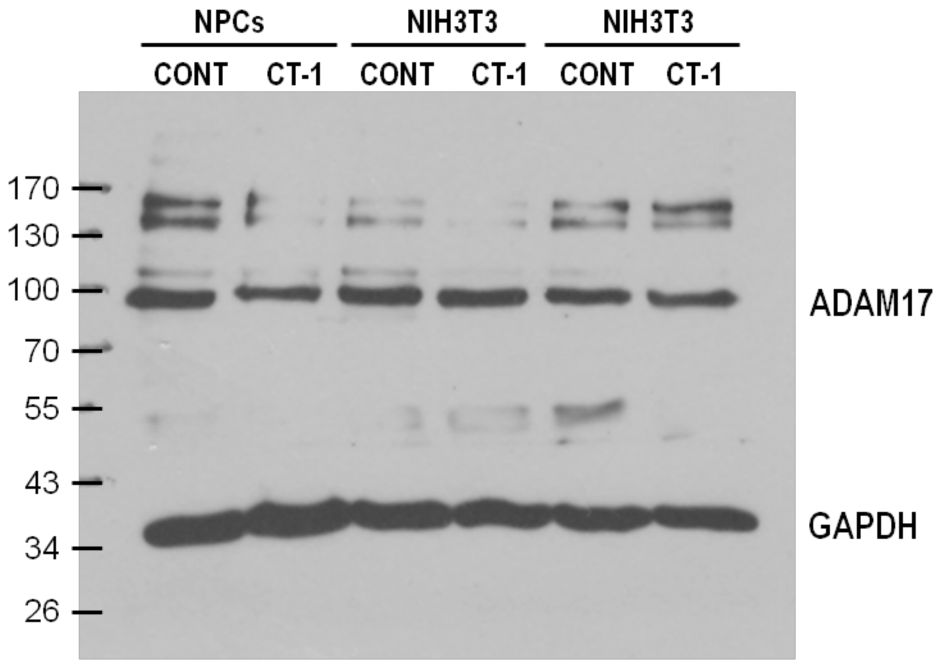

ADAM17 expression for Fig.1D

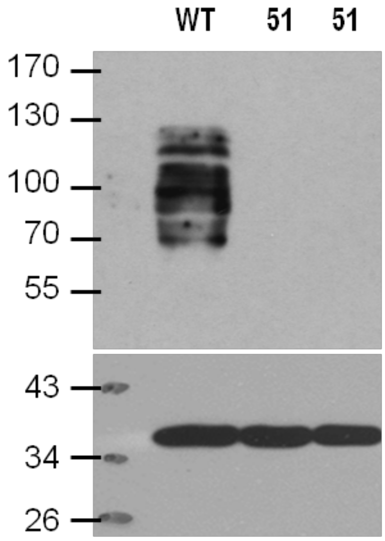

ADAM10 is not expressed in 293T cell line #51 for Fig.2A.

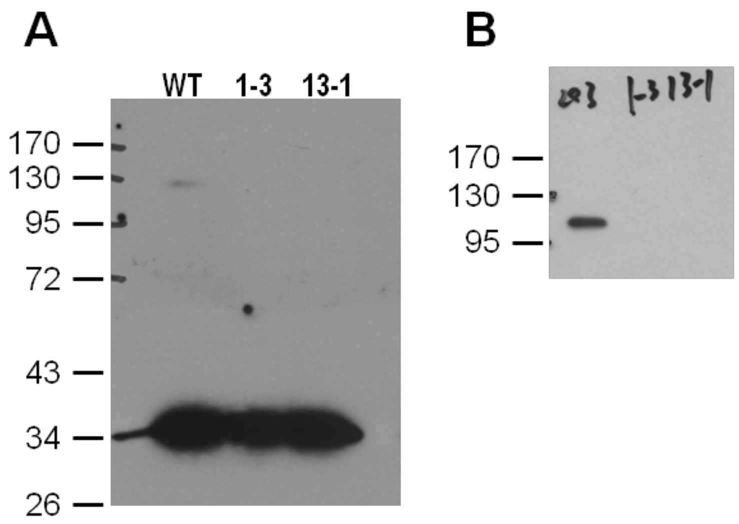

ADAM17 is not expressed in 293T cell lines #1-3 and #13-1 for Fig.2A. The tope part of the blot was washed and re-exposed for longer period to observe ADAM17 expression in WT cells.

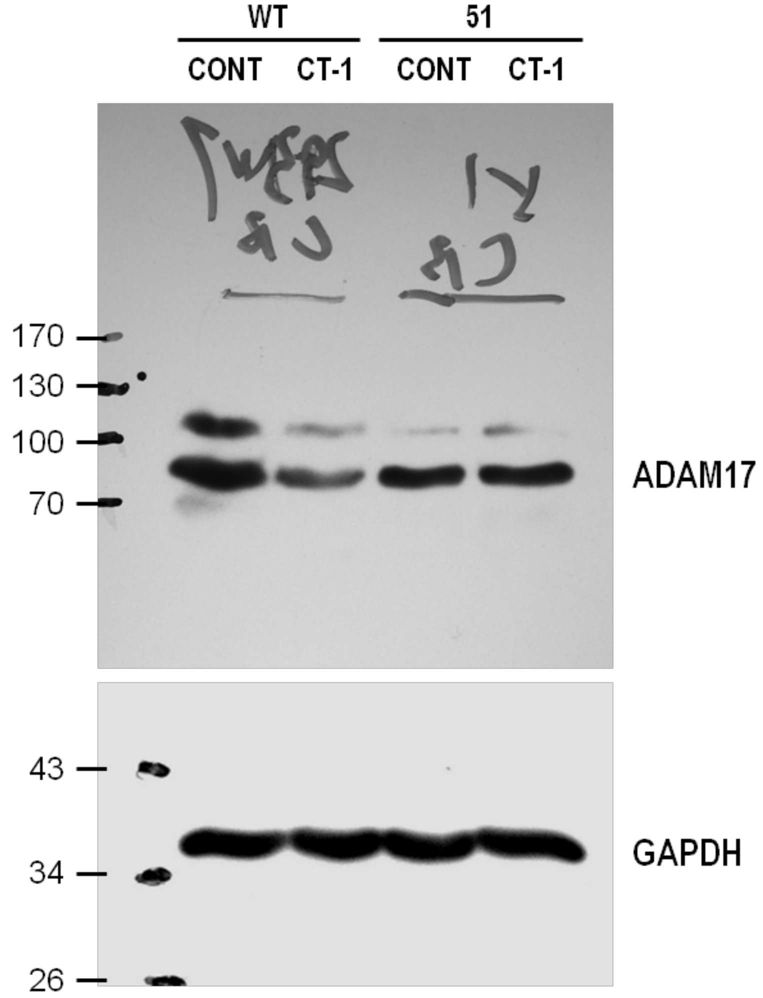

ADAM17 expression in WT and ADAM10-KO cells for Fig.2B.

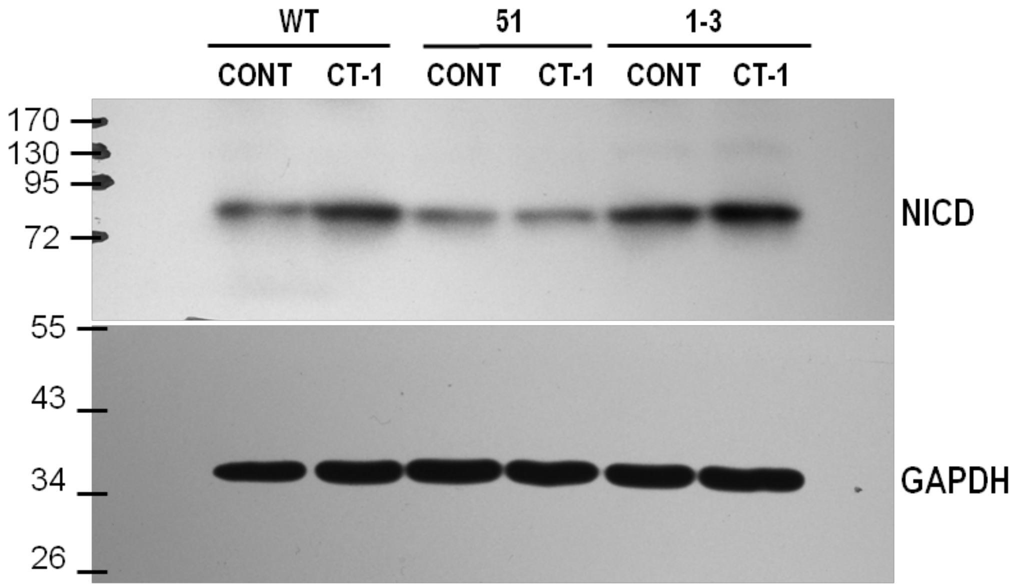

NICD in WT and ADAM10 or ADAM17 KO cells for Fig.2B.

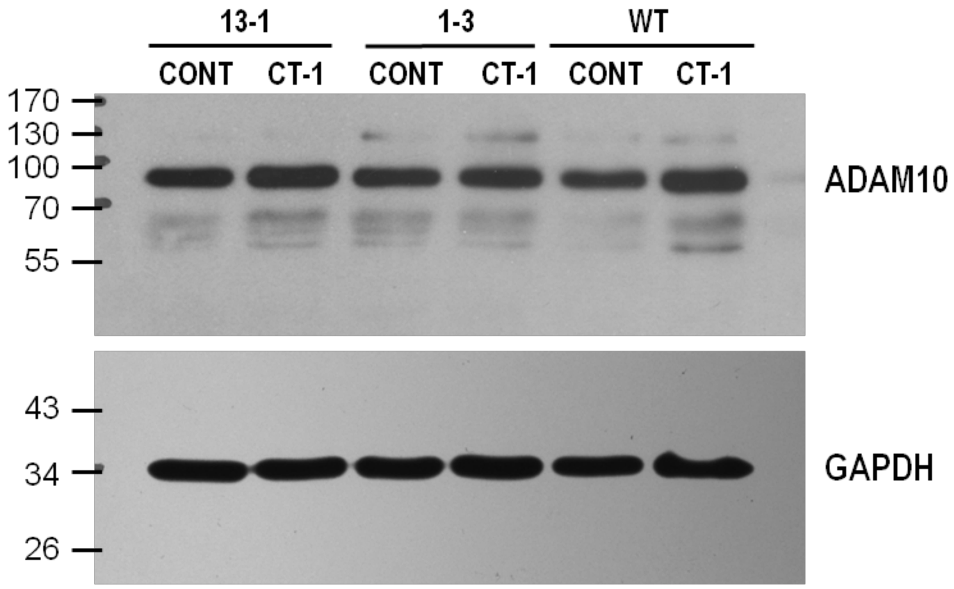

ADAM10 in WT and ADAM17 KO cell lines for Fig.2B

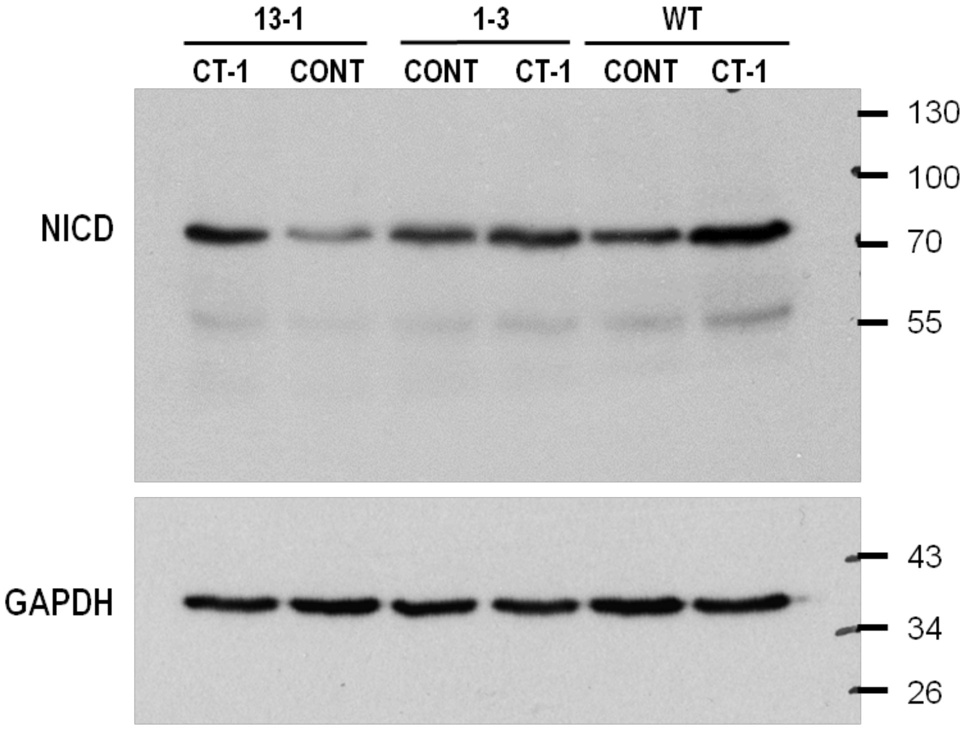

NICD in WT and ADAM17 KO cell lines for Fig.2B

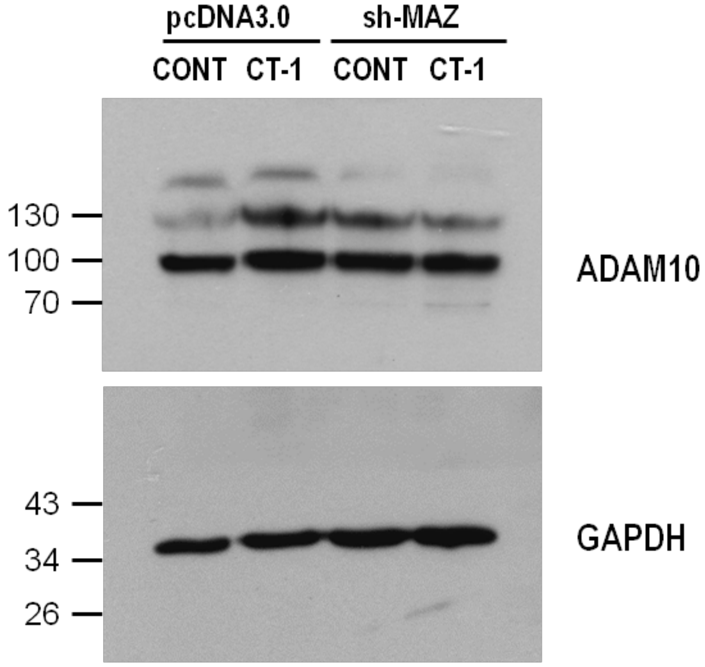

ADAM10 in MAZ knockdown NPCs for Fig.4A.

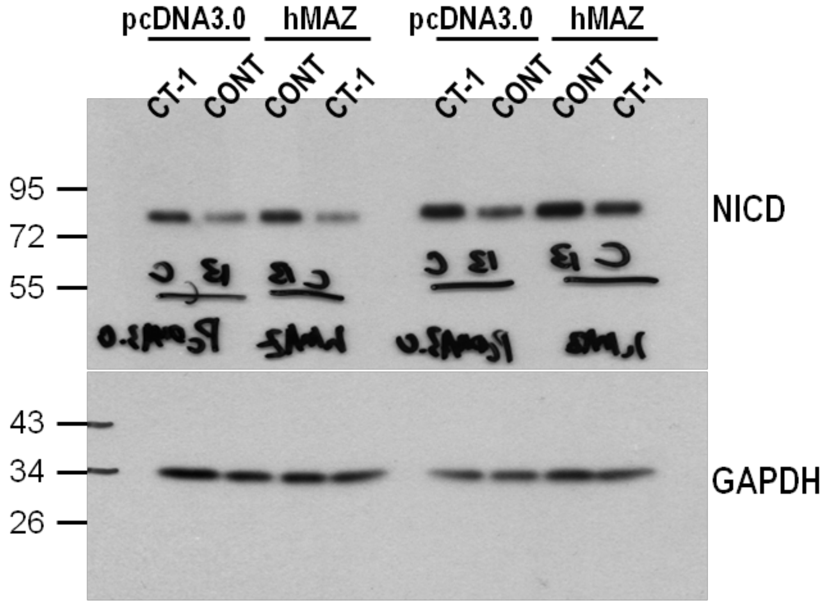

NICD expression in hMAZ overexpressing NPCs for Fig.4B.

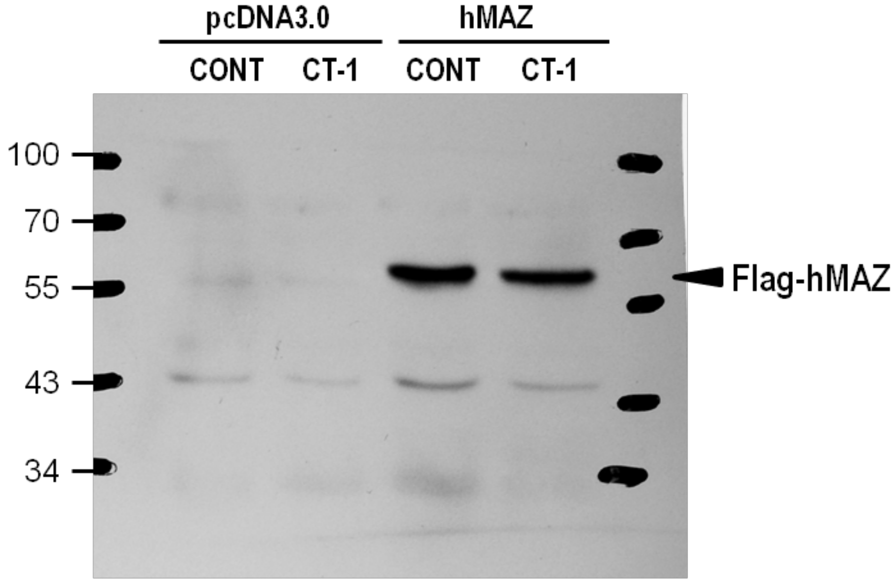

hMAZ expression in NPCs for Fig.4B.

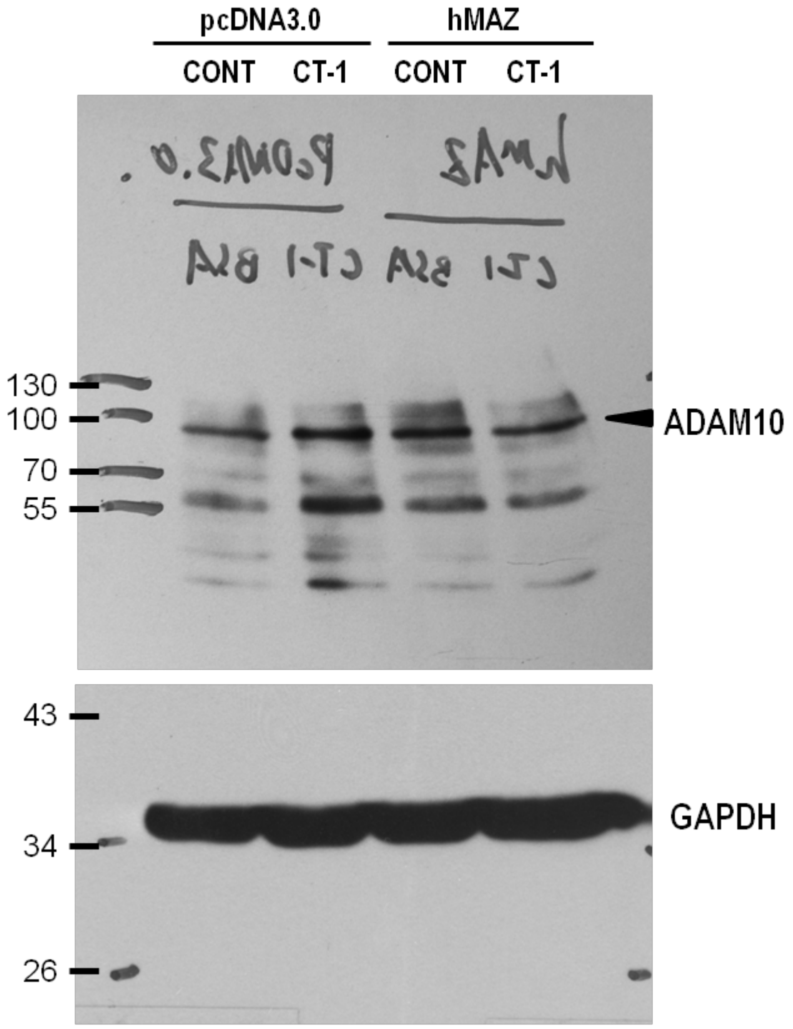

ADAM10 expression in hMAZ over-expressing NPCs for Fig.4B.

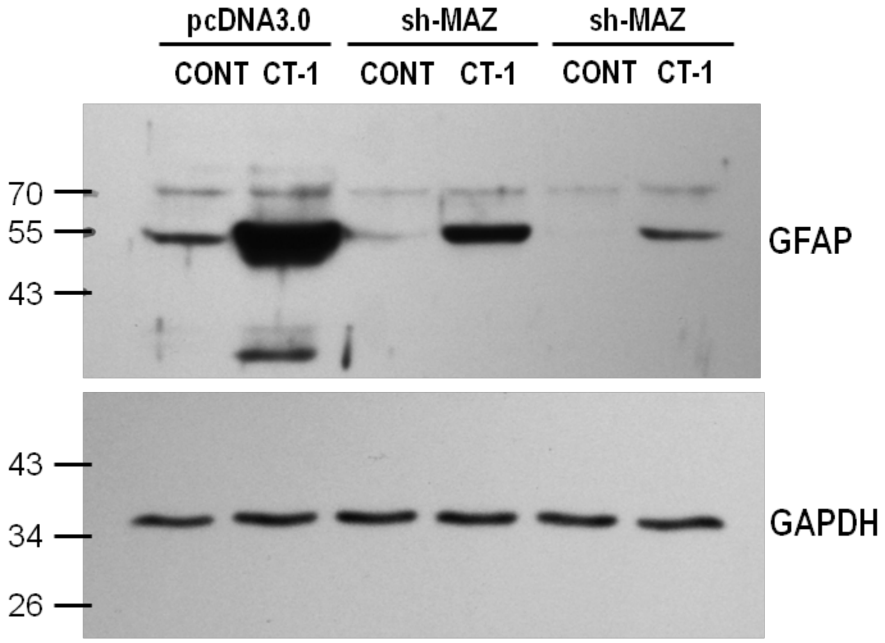

GFAP expression in MAZ knockdown NPCs for Fig.4C.

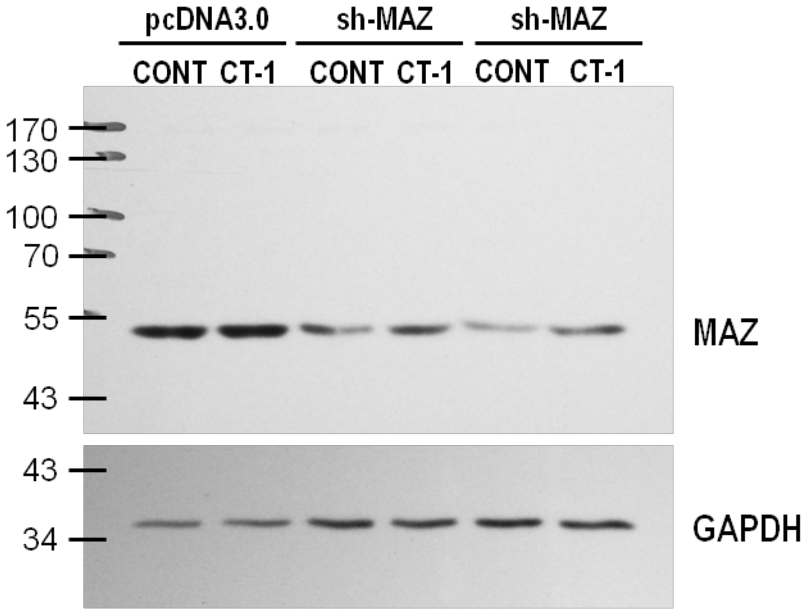

MAZ knockdown in NPCs for Fig.4A.

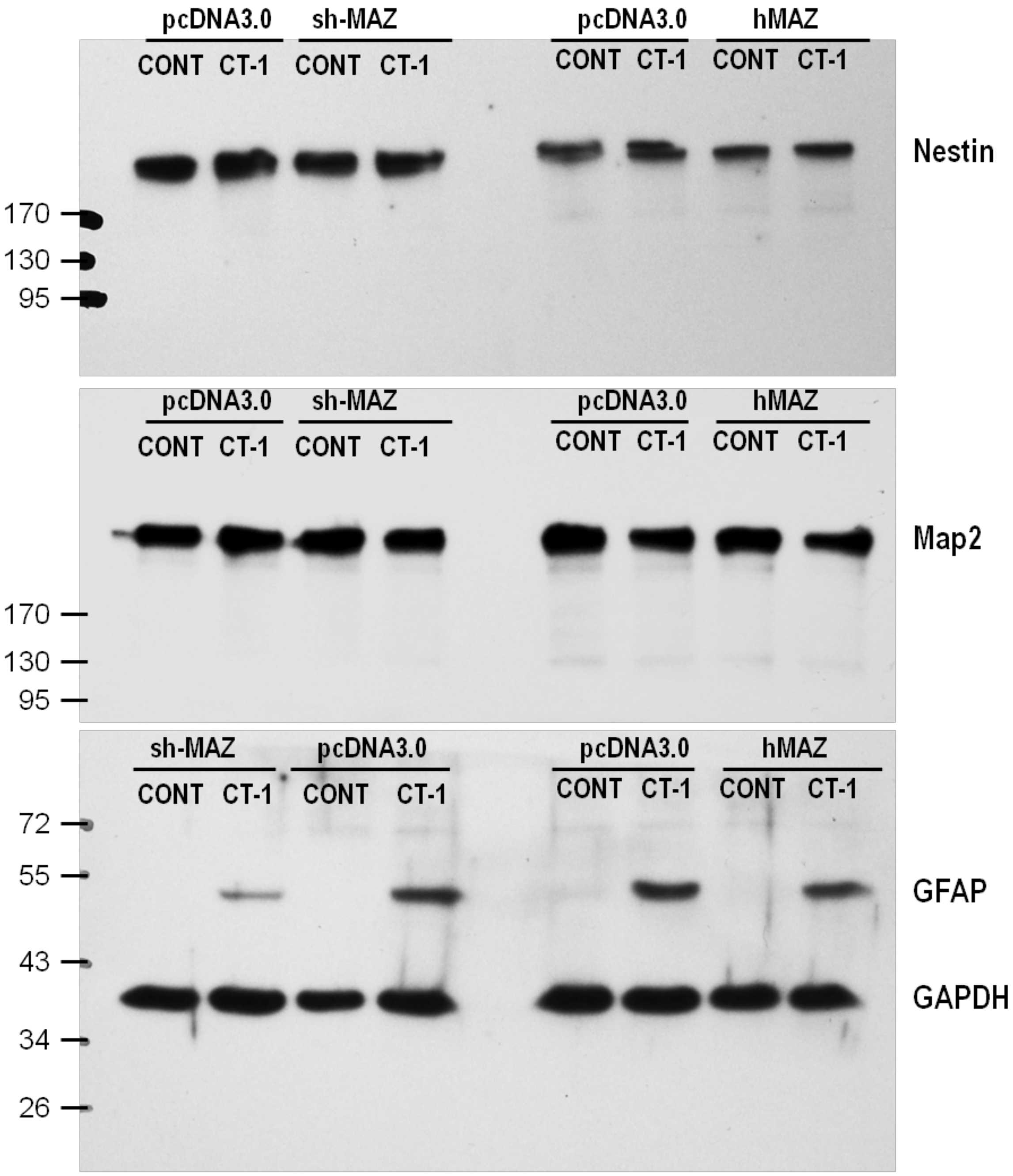

Nestin, Map2, and GFAP expression in MAZ knockdown or hMAZ overexpressing NPCs for Fig.4C&4D.

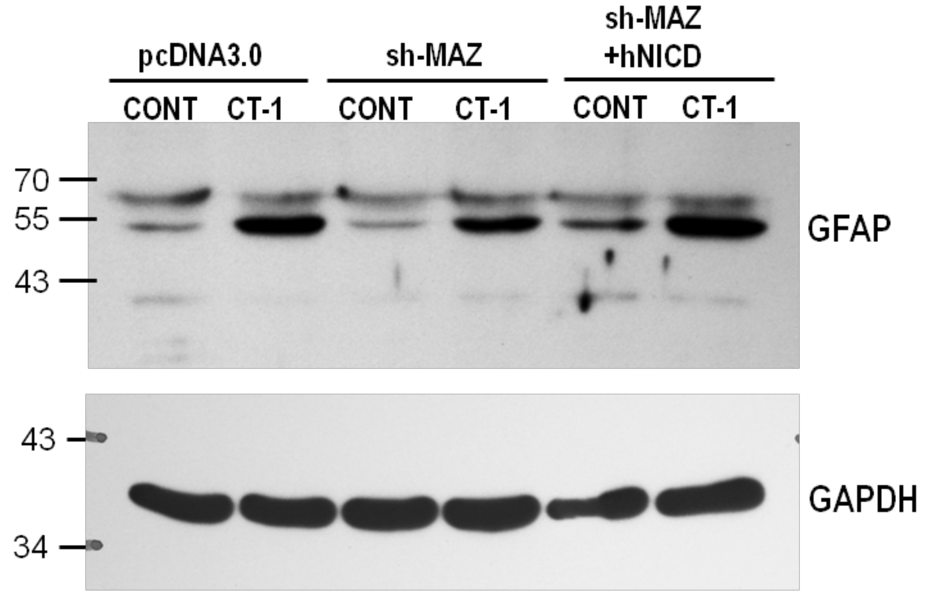

GFAP expression in MAZ knockdown or hNICD overexpressing NPCs for Fig.4E.

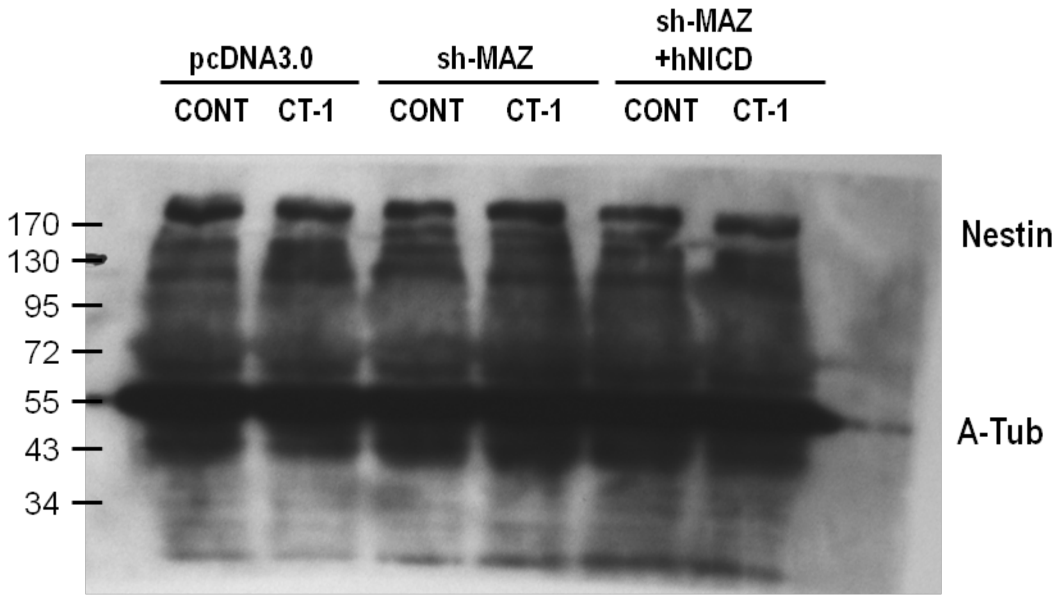

Nestin expression in MAZ knockdown or or hNICD overexpressing NPCs for Fig.4E.

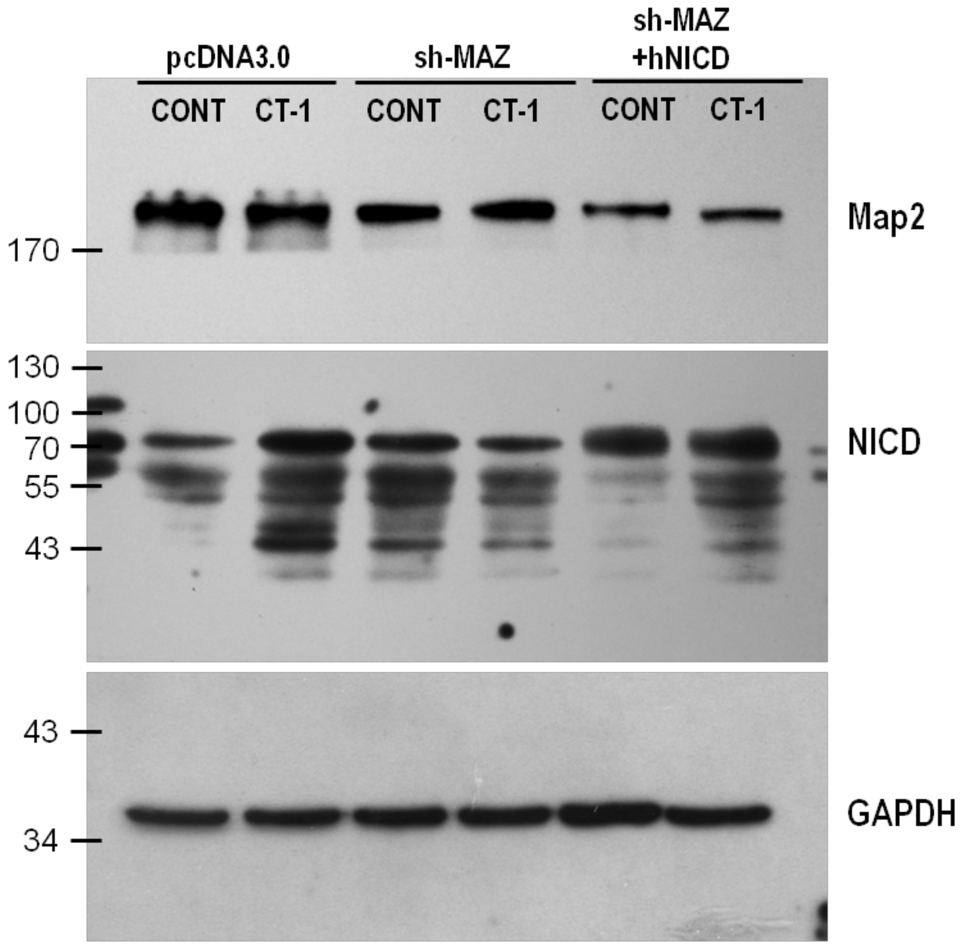

Map2 and NICD expression in MAZ knockdown or or hNICD overexpressing NPCs for Fig.4E.

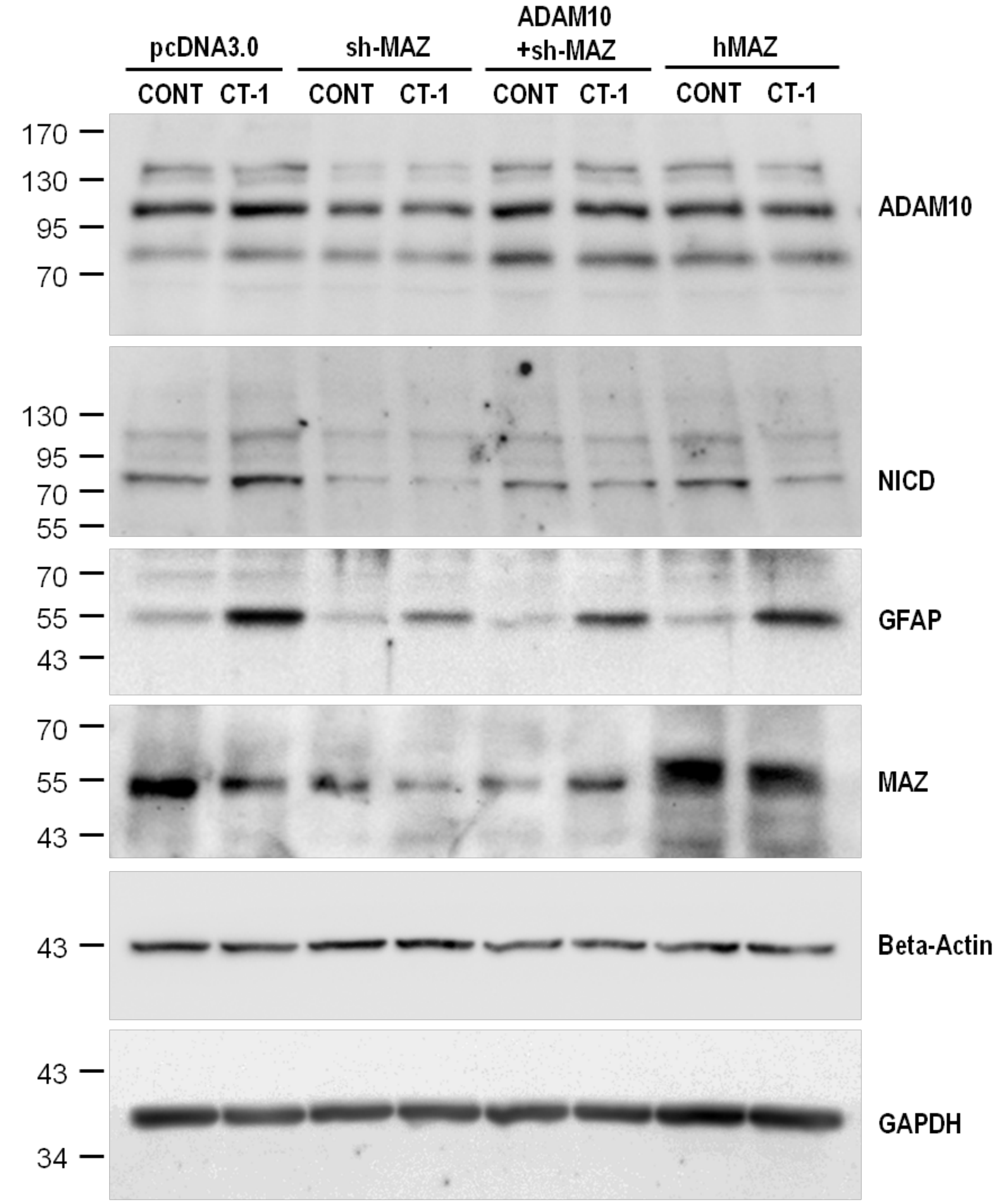

ADAM10, NICD, GFAP, MAZ, β-Actin, GAPDH expression for Figure S2.
